# Chemokine Receptor Redundancy and Specificity Are Context Dependent

**DOI:** 10.1016/j.immuni.2019.01.009

**Published:** 2019-02-19

**Authors:** Douglas P. Dyer, Laura Medina-Ruiz, Robin Bartolini, Fabian Schuette, Catherine E. Hughes, Kenneth Pallas, Francesca Vidler, Megan K.L. Macleod, Christopher J. Kelly, Kit Ming Lee, Christopher A.H. Hansell, Gerard J. Graham

**Affiliations:** 1 Chemokine Research Group, Institute of Infection, Immunity and Inflammation, College of Medical, Veterinary and Life Sciences, University of Glasgow, Glasgow G12 8TT, UK

**Keywords:** chemokines, inflammation, monocytes, dendritic cells, receptors, mouse models

## Abstract

Currently, we lack an understanding of the individual and combinatorial roles for chemokine receptors in the inflammatory process. We report studies on mice with a compound deletion of *Ccr1*, *Ccr2*, *Ccr3*, and *Ccr5*, which together control monocytic and eosinophilic recruitment to resting and inflamed sites. Analysis of resting tissues from these mice, and mice deficient in each individual receptor, provides clear evidence for redundant use of these receptors in establishing tissue-resident monocytic cell populations. In contrast, analysis of cellular recruitment to inflamed sites provides evidence of specificity of receptor use for distinct leukocyte subtypes and no indication of comprehensive redundancy. We find no evidence of involvement of any of these receptors in the recruitment of neutrophils or lymphocytes to resting or acutely inflamed tissues. Our data shed important light on combinatorial inflammatory chemokine receptor function and highlight *Ccr2* as the primary driver of myelomonocytic cell recruitment in acutely inflamed contexts.

## Highlights

• Mice deficient in CCR1, CCR2, CCR3, and CCR5 (iCCRs) develop normally
• iCCRs redundantly establish resting tissue-resident myelomonocytic cell populations
• CCR2 dominates in controlling monocyte recruitment in acute inflammation
• iCCRs are not involved in neutrophil or lymphocyte recruitment in acute inflammation

## Teaser

CCR1, CCR2, CCR3, and CCR5 (iCCRs) are dominant regulators of myelomonocytic cell recruitment in inflammatory disease. Here, Dyer et al. have deleted the genomic locus incorporating the iCCRs in mice and demonstrate redundancy of iCCR involvement in establishing tissue-resident myelomonocytic cell populations yet specificity of iCCR use in acute inflammation.

## Introduction

Leukocyte migration is regulated by chemokines (Rot and von Andrian, 2004), which are characterized by conserved cysteine motifs and which exert their effects by binding to 7-transmembrane-spanning receptors (Bachelerie et al., 2014a). Chemokines and their receptors are broadly defined as being inflammatory or homeostatic according to the contexts in which they function (Mantovani, 1999, Zlotnik and Yoshie, 2000), and their biology is further fine-tuned by stromally expressed atypical chemokine receptors (Bachelerie et al., 2014b, Nibbs and Graham, 2013).

Chemokines and their receptors have emerged as prominent players and key therapeutic targets in a wide range of immune and inflammatory disorders (Griffith et al., 2014, Proudfoot, 2002, Viola and Luster, 2008). However, despite extensive research, no antagonists of inflammatory chemokine receptors have been licensed for use in inflammatory diseases (Bachelerie et al., 2014a, Schall and Proudfoot, 2011), and this is partly due to the complexity of inflammatory chemokine and chemokine receptor biology and biochemistry. For example, inflammatory chemokine receptors display promiscuous ligand binding (Bachelerie et al., 2014a), and the chemokines in turn bind to multiple different chemokine receptors. It is unclear to what extent this represents biological redundancy (Mantovani, 1999) or whether there are discrete signals triggered by different chemokines through individual chemokine receptors, and this remains a controversial area (Schall and Proudfoot, 2011, Steen et al., 2014). Further complicating our understanding of the chemokine-driven inflammatory response is the fact that individual leukocyte subsets appear to simultaneously express multiple inflammatory chemokine receptors (Haringman et al., 2006, Tacke et al., 2007, Weber et al., 2000). It is therefore currently not possible to say with any degree of certainty which chemokine receptors monocytes, for example, would use to migrate to an inflammatory site.

We have been studying four of the inflammatory chemokine receptors: *Ccr1*, *Ccr2*, *Ccr3*, and *Ccr5* (henceforth referred to as i*Ccr*s). Evolutionarily, *Ccr2* is the oldest of these four receptors (Nomiyama et al., 2011). From it, the others have emerged through gene duplication to occupy a discrete and tight chromosomal locus (170 kb) on mouse chromosome 9 (human chromosome 3). Together, the i*Ccr*s are responsible for myelomonocytic cell recruitment to inflamed sites (Shi and Pamer, 2011). However, the combinatorial, and in some cases the individual, roles for these receptors in leukocyte recruitment are currently unclear, and this is an issue of controversy and confusion within the field (Gautier et al., 2009, Sandblad et al., 2015, Soehnlein et al., 2013, Tacke et al., 2007, Weber et al., 2000). Overall, we lack an integrated understanding of how these four receptors regulate myelomonocytic cell recruitment during inflammation. The issue of redundancy versus specificity of inflammatory chemokine receptor function also remains unresolved for the i*Ccr*s (Mantovani, 1999, Schall and Proudfoot, 2011). A further hindrance to studies in this area is the close genomic association of the genes encoding the i*Ccr*s. Therefore, generating compound-receptor-deficient mice to examine combinatorial receptor function has been impractical.

Here, we have deleted the entire i*Ccr* locus (iCCR-deficient mice) and have examined the recruitment of leukocytes to both resting and acutely inflamed sites between these mice and both wild-type (WT) and single-receptor-deficient mice. The iCCR-deficient mice are viable and healthy but display profound defects in inflammatory leukocyte recruitment. Our results provide evidence for both redundancy and specificity in the function of the i*Ccr*s and highlight the primacy of *Ccr2* as a recruiter of monocytic cells to acutely inflamed sites.

## Results

### Deletion of the i*Ccr* Locus Is Not Associated with Developmental Abnormalities

The i*Ccr*s are contained within a 170 kb genomic locus situated at the telomeric end of mouse chromosome 9 (Figure S1A). This is a “pristine” locus, and it contains no other genes (with the exception of a poorly characterized and weakly conserved putative chemokine receptor, *Ccr1l1* [Nomiyama et al., 2011], which is absent from the human genome), thus ensuring that excision of this locus affects only the i*Ccr*s. We deleted the locus by inserting LoxP sites at its 5′ and 3′ extremes and inducing Cre-mediated excision in embryonic stem (ES) cells (Figure S1A). Heterozygous mice were generated from these ES cells and bred to homozygosity. Homozygous mice were born at the expected Mendelian frequency from heterozygote crosses (Figure S1B) and were healthy and fertile. Deletion of the four chemokine receptors was further confirmed by PCR analysis of expression in peripheral-blood leukocytes of heterozygous offspring, which revealed 50% of WT expression of *Ccr1*, *Ccr2*, *Ccr3*, and *Ccr5* but unaltered expression of *Cxcr2*, which sits outside the targeted locus (Figure S1C). T cells from iCCR-deficient mice displayed identical responses to WT cells after Cxcl10 treatment (Figure S1D), and monocyte-derived macrophages responded identically to Cx3cL1 treatment (data not shown), indicating that multi-receptor deletion does not alter responses through other non-deleted chemokine receptors. iCCR-deficient embryos appeared grossly normal (Figure S1E), and no differences were noted in numbers of fetal liver monocytes (Figures S1Fi and S1Fii) or cKit^+^ hematopoietic progenitor cells (Figures S1Gi and S1Gii).

Thus, the i*Ccr*s are not essential for development or postnatal survival, and deletion of the locus is not associated with any gross developmental abnormalities.

### iCCR-Deficient Blood Displays *Ccr2*-like Monocytopenia

**Figure 1:**
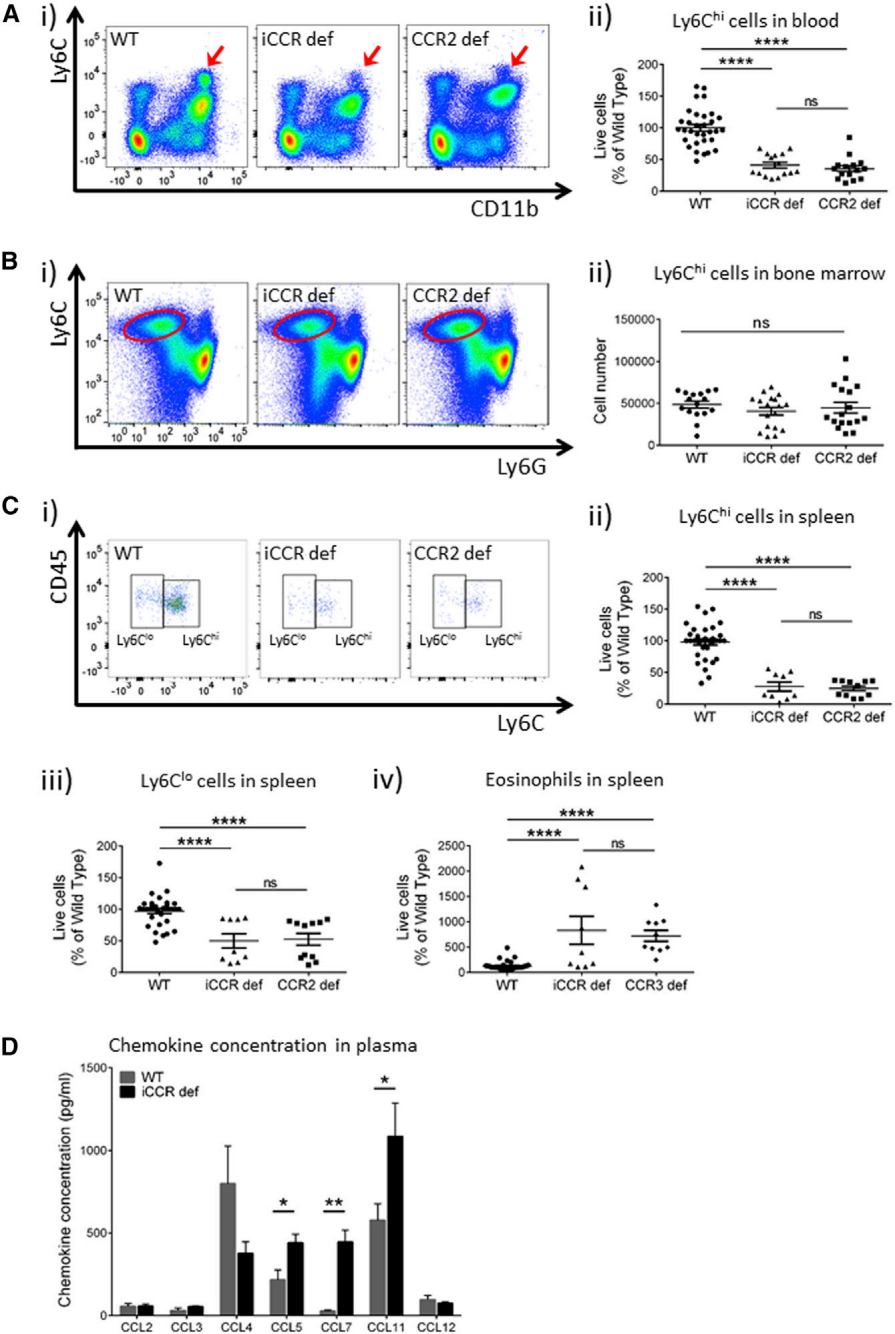
iCCR-Deficient Mice Display Resting Blood and Spleen Defects (A) (i) Flow-cytometric analysis of Ly6C^hi^ and CD11b^+^ cells (arrows) in the blood of WT, iCCR-deficient, and CCR2-deficient mice. (ii) Quantification of Ly6C^hi^ cells in the blood of WT (n = 32), iCCR-deficient (n = 15), and CCR2-deficient (n = 15) mice. (B) (i) Flow-cytometric analysis of Ly6C^hi^ cells in the bone marrow of WT, iCCR-deficient, and CCR2-deficient mice. (ii) Quantification of Ly6C^hi^ cells in the bone marrow of WT (n = 16), iCCR-deficient (n = 18), and CCR2-deficient (n = 17) mice. (C) (i) Flow-cytometric analysis of CD45^+^ and Ly6C^hi^ cells (in the CD11c^−^MHCII^−^ gate) in the spleen of WT, iCCR-deficient, and CCR2-deficient mice, as well as quantification of (ii) Ly6C^hi^ cells, (iii) Ly6C^lo^ cells, and (iv) eosinophils in the spleen of WT (n = 33), iCCR-deficient (n = 9), and CCR2-deficient (n = 11) mice (and eosinophils in CCR3-deficient mice [n = 10]). (D) Luminex analysis of chemokine concentrations in the plasma of WT mice (gray bars) and iCCR-deficient mice (black bars). All numerical data in (Aii), (Bii), (Cii)–(Civ), and (D) are presented as mean + SEM. ^∗^p < 0.05; ^∗∗^p < 0.01; ^∗∗∗∗^p < 0.0001, n.s., not significant. All experiments are representative of at least three repeat experiments, which were analyzed by one-way ANOVA on log-transformed data. Each data point represents a measurement from a single mouse. Please also see Figures S1, S2, S5, and S7 and Table S1.

Analysis of peripheral-blood leukocytes demonstrated a marked reduction in the numbers of circulating Ly6C^hi^CD11b^+^ monocytic cells in resting iCCR-deficient mice compared with WT mice (Figure 1Ai) but no alteration in numbers of cells from any other tested hematopoietic lineages (Figure S3A). Quantification of the reduction in monocytic cell numbers in iCCR-deficient blood demonstrated that this reduction was specifically for Ly6C^hi^ inflammatory monocytes (60% reduction; Figure 1Aii and Figure S3A), and no significant differences were detected in the numbers of circulating Ly6C^lo^ cells (Table S1 and Figure S3A). As previously reported (Serbina and Pamer, 2006, Tsou et al., 2007), CCR2-deficient mice displayed an identical reduction in circulating Ly6C^hi^ monocyte numbers (Figures 1Ai and 1Aii), indicating that, in terms of peripheral-blood leukocyte content, iCCR-deficient mice essentially phenocopied CCR2-deficient mice. There was a modest reduction in Ly6C^hi^ monocyte numbers in the blood of CCR1-deficient mice (Table S1), which reflected the reported reduction in hematopoietic progenitor cell numbers in CCR1-deficient peripheral blood (Gao et al., 1997). However, the relevance of this is unclear because there was no further reduction in Ly6C^hi^ monocyte numbers in the blood of iCCR-deficient, compared with CCR2-deficient, mice. No significant differences in circulating monocyte numbers (or indeed in numbers of any other tested circulating hematopoietic lineages) were seen in CCR3-deficient or CCR5-deficient mice (Table S1). Thus, our data demonstrate that monocyte egress from bone marrow to the resting circulation is fully and non-redundantly dependent on *Ccr2*.

In contrast to previous studies of CCR2-deficient mice (Serbina and Pamer, 2006, Tsou et al., 2007), we failed to detect any corresponding increase in CD11b^+^Ly6C^hi^ cell numbers in either iCCR-deficient or CCR2-deficient bone marrow (Figures 1Bi and 1Bii). In addition, no differences were detected in any other tested hematopoietic lineages in the bone marrow from iCCR-deficient mice (Figure S3B), and no differences were detected in any of the other single-receptor-deficient mice (data not shown). Analysis of the spleen (Figure 1Ci) revealed a selective reduction in numbers of both Ly6C^hi^ and Ly6C^lo^ monocytes in iCCR-deficient mice (Figures 1Cii and 1Ciii), which again phenocopied CCR2-deficient mice. A modest but significant decrease in splenic Ly6C^hi^ monocyte numbers was seen in CCR1-deficient mice (Figure 1), and, in this case, a reduction was also seen in CCR5-deficient mice (Table S1). Again, there was no apparent redundancy at play here because the extent of impairment of Ly6C^hi^ monocyte recruitment to the spleen was the same in iCCR-deficient and CCR2-deficient mice. No differences in monocytic recruitment were noted in CCR3-deficient mice (Table S1). With the exception of a significant increase in eosinophils (which, as reported previously [Humbles et al., 2002], is also seen in CCR3-deficient spleens; Figure 1Civ), no other hematopoietic lineages were altered in the iCCR-deficient spleens (data not shown) or in the spleens of other receptor-deficient mice studied.

**Figure 2:**
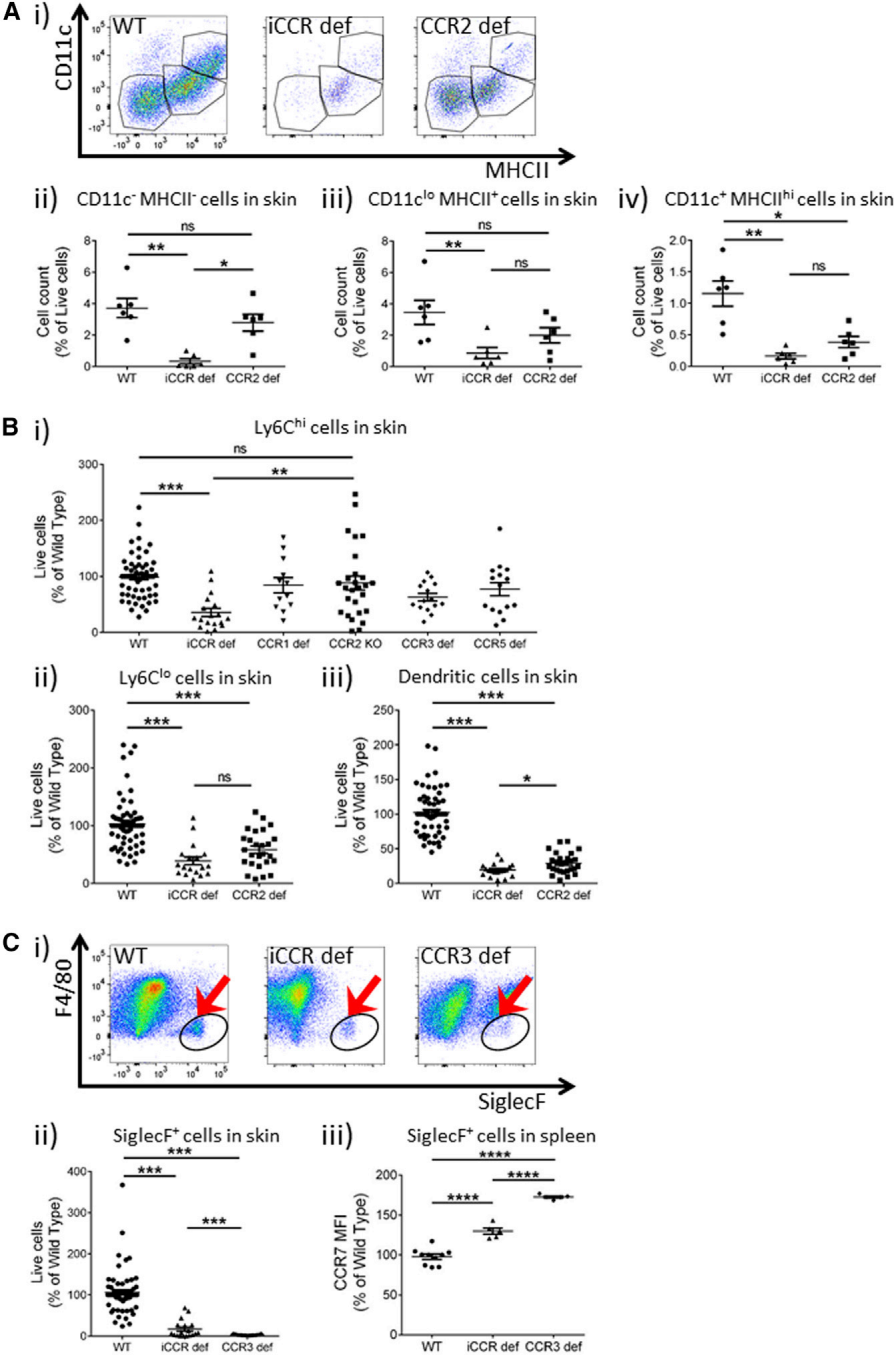
iCCR-Deficient Mice Display Resting Defects in Myelomonocytic Cell Recruitment to Skin (A) (i) Flow-cytometric assessment of CD11c and MHCII expression among CD45^+^CD11b^+^ cells from WT, iCCR-deficient, and CCR2-deficient mice. Numbers of (ii) CD11c^−^MHCII^−^, (iii) CD11c^lo^MHCII^+^, and (iv) CD11c^+^MHCII^hi^ cells are shown as a percentage of live cells in WT (n = 6), iCCR-deficient (n = 6), and CCR2-deficient (n = 6) mice. (B) (i) Flow-cytometric assessment of myelomonocytic cells gated for (i) Ly6C^hi^, (ii) Ly6C^lo^, and (iii) dendritic cells (WT, n = 54; iCCR deficient, n = 15; CCR1 deficient, n = 12; CCR2 deficient, n = 22; CCR3 deficient, n = 15; CCR5 deficient, n = 15). (C) (i) Flow-cytometric assessment (eosinophils indicated by arrows) and (ii) quantification of eosinophil numbers (WT, n = 54; iCCR deficient, n = 15; CCR3 deficient, n = 15). (iii) Analysis of *Ccr7* expression on splenic eosinophils from WT, CCR3-deficient, and iCCR-deficient mice. All numerical data in (Aii)–(Aiv), (B), (Cii), and (Ciii) are presented as mean + SEM. ^∗^p < 0.05; ^∗∗^p < 0.01; ^∗∗∗^p < 0.001; ^∗∗∗∗^p < 0.0001; n.s., not significant. Data in (A) are representative of at least three repeat experiments, and data in (B) and (C) are compiled from at least three independent experiments. In all cases, data were analyzed by one-way ANOVA on log-transformed data. Each data point represents a measurement from a single mouse. Please also see Figures S3, S6, and S7 and Table S1.

Luminex analysis (Figure 1D) of WT and iCCR-deficient plasma revealed significantly higher concentrations of *Ccl5* (*Ccr1*, *Ccrl3*, and *Ccrl5* ligands), *Ccl7* (*Ccl1*, *Ccl2*, *Ccl3*, and *Ccl5* ligands), and *Ccl11* (*Ccr3* ligand) in iCCR-deficient mice than in WT mice, suggesting that in WT mice, these chemokines are actively scavenged at rest by their cognate receptors, which indicates that these receptors are functional in resting cell recruitment. This suggests that *Ccl11* plays a prominent role in basal eosinophil migration into resting tissues (Figure 2). Furthermore, the specific increase in *Ccl*7 concentrations indicated that it is likely to be the primary *Ccr2* ligand involved in monocyte egress from the bone marrow and entry into resting peripheral tissues. This finding is in agreement with previous studies (Bardina et al., 2015, Tsou et al., 2007) that demonstrated a crucial functional role for *Ccl7* in monocyte egress from bone marrow. The elevated concentrations of *Ccl5* are also compatible with a role for *Ccr1* in contributing to the efficiency of bone marrow egress of monocytic cells. The issue of chemokine use by resting tissues is also addressed in the Discussion.

Thus, iCCR-deficient peripheral blood is characterized by a substantial reduction in Ly6C^hi^ monocyte numbers and essentially recapitulates the circulatory phenotype observed in CCR2-deficient mice. We conclude that Ly6C^hi^ monocyte egress from bone marrow is fully and non-redundantly dependent on *Ccr2*.

### Redundancy Is Evident in Receptor Use for Recruitment of Myelomonocytic Cell Populations to Resting Skin

Next, we examined the leukocyte content of resting iCCR-deficient skin. Flow-cytometric analysis of skin from adult WT, iCCR-deficient, and CCR2-deficient mice revealed a reduction in the overall CD45^+^ cell content in both iCCR-deficient and CCR2-deficient skin (data not shown), which was not seen in other single-receptor-deficient mice. Broad-based flow-cytometric assessment of discrete CD11c^−^MHCII^−^, CD11c^lo^MHCII^+^, and CD11c^hi^MHCII^hi^ populations revealed (Figures 2Ai–2Aiv) substantial reduction in all three populations in iCCR-deficient mice, but significant reduction was seen only in CD11c^hi^MHCII^hi^ dendritic cells in CCR2-deficient mice (Figure 2Aiv).

Further myelomonocytic cell subtyping revealed a marked reduction (70%) in the size of the Ly6C^hi^ monocytic population in iCCR-deficient mice, whereas, no significant differences were seen in the Ly6C^hi^ population in any of the single-receptor-deficient mice, including CCR2-deficient mice (Figure 2Bi and Table S1). Notably, this analysis is powered to detect a 25% variation in iCCR-deficient cell numbers compared with WT numbers, and thus no single receptor can account for the substantial reduction in the Ly6C^hi^ population observed in iCCR-deficient mice. These data therefore clearly indicate redundancy in receptor involvement in recruitment of Ly6C^hi^ cells or their precursors to resting skin.

Reductions of 40%–60% were noted for Ly6C^lo^ cells in both iCCR-deficient and CCR2-deficient mice (Figure 2Bii), and although both genotypes displayed a reduction in dendritic cell numbers (Figure 2Biii), this was significantly greater in iCCR-deficient mice (80% reduction). Finally, resident eosinophil numbers were reduced in iCCR-deficient skin closely phenocopying CCR3-deficient mice, although the reduction in CCR3-deficient mice was consistently more profound than that seen in iCCR-deficient mice (Figures 2Ci and 2Cii). We detected higher expression of CCR7 on splenic eosinophils from CCR3-deficient and iCCR-deficient mice than on those from WT mice, and the increase was significantly greater in CCR3-deficient than in iCCR-deficient eosinophils (Figure 2Ciii). This suggests a possible mechanism for the more comprehensive depletion of eosinophils in the CCR3-deficient mice, i.e., that they are more competent for tissue egress. In contrast to iCCR-deficient, CCR2-deficient, and CCR3-deficient mice, CCR1- and CCR5-deficient mice displayed no reductions in the size of any of the measured leukocyte populations (Table S1). No differences were detected in any lymphoid subtype in iCCR-deficient resting skin (Figure S4A).

Overall, this analysis demonstrates clear redundancy in the involvement of the receptors within the i*Ccr* locus in establishing the resting skin Ly6C^hi^ population.

### Receptor Involvement in Resting Leukocyte Recruitment Varies between Tissues

**Figure 3:**
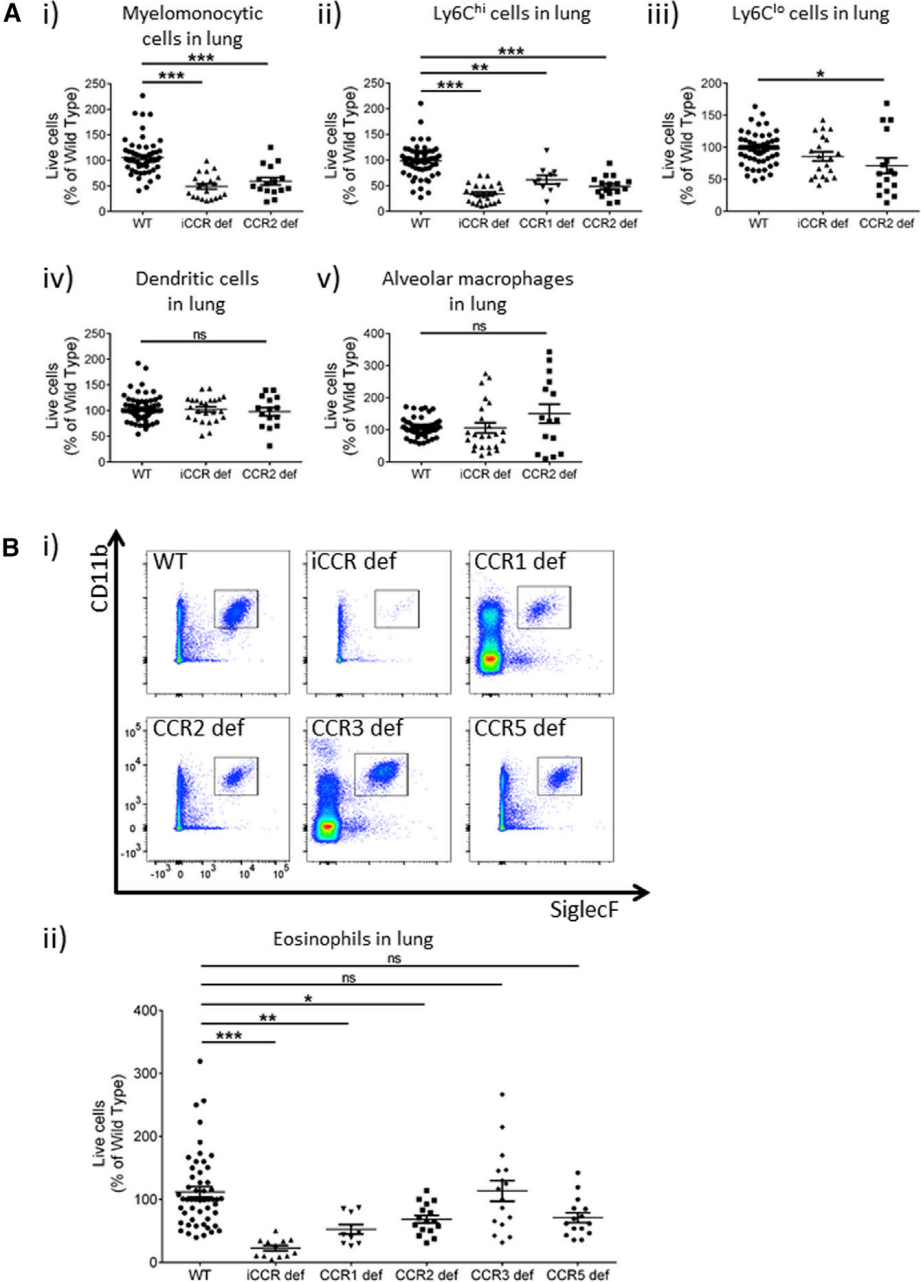
Resting Myelomonocytic Cell Content of Lung (A) Flow-cytometric analysis of (i) total myelomonocytic cells, (ii) Ly6C^hi^ cells, (iii) Ly6C^lo^ cells, (iv) dendritic cells, and (v) alveolar macrophages (WT, n = 62; iCCR deficient, n = 20; CCR2 deficient, n = 16; CCR1 deficient, n = 10). (B) Flow-cytometric (i) and quantitative (ii) analysis of eosinophil numbers in all mouse strains (WT, n = 52; iCCR deficient, n = 12; CCR2 deficient, n = 16; CCR1 deficient, n = 10; CCR3 deficient, n = 16; CCR5 deficient, n = 15). All numerical data in (A) and (Bii) are presented as mean + SEM. ^∗^p < 0.05; ^∗∗^p < 0.01; ^∗∗∗^p < 0.001; n.s., not significant. Data are compiled from at least three independent experiments. In all cases, data were analyzed by one-way ANOVA on log-transformed data. Each data point represents a measurement from a single mouse. Please also see Figures S3, S6, and S7 and Table S1.

We performed a similar analysis of resident leukocytes in the lungs and (as shown in Figure 3Ai) again observed significant reduction in total monocyte and macrophage numbers in both iCCR-deficient and CCR2-deficient resting mice. In-depth phenotyping revealed a strong depletion of Ly6C^hi^ monocytes in both iCCR-deficient and CCR2-deficient mice (Figure 3Aii). A lesser, but significant, depletion was noted in *CCR1*-deficient mice, but no differences were seen in CCR3-deficient or CCR5-deficient mice (Table S1). No detectable changes were seen in Ly6C^lo^ cells (apart from a slight reduction in CCR2-deficient mice, but not in iCCR-deficient mice), dendritic cells (Figures 3Aiii and 3Aiv and Table S1), or any of the other myelomonocytic cell populations examined. No differences were detected in alveolar macrophage numbers (Figure 3Av and Table S1). Finally, a profound depletion of eosinophils was seen in the iCCR-deficient lungs (Figures 3Bi and 3Bii). In keeping with previous reports (Humbles et al., 2002, Pope et al., 2005), we did not observe depletion of eosinophil numbers in CCR3-deficient lungs but did detect significant reductions in numbers in CCR1-deficient and CCR2-deficient lungs (Figures 3Bi and 3Bii and Table S1). Again, no differences were noted in the size of any lymphocyte populations (Figure S4B) in iCCR-deficient mice, and no alterations in any of the other measured populations were detectable in CCR1-deficient or CCR5-deficient mice (Table S1).

Thus, resting iCCR-deficient tissues are characterized by comprehensive depletion of a range of myelomonocytic cell subtypes, which varies according to tissue type.

### Resting Skin and Lung Display Differential Patterns of i*Ccr* Ligand Use

**Figure 4:**
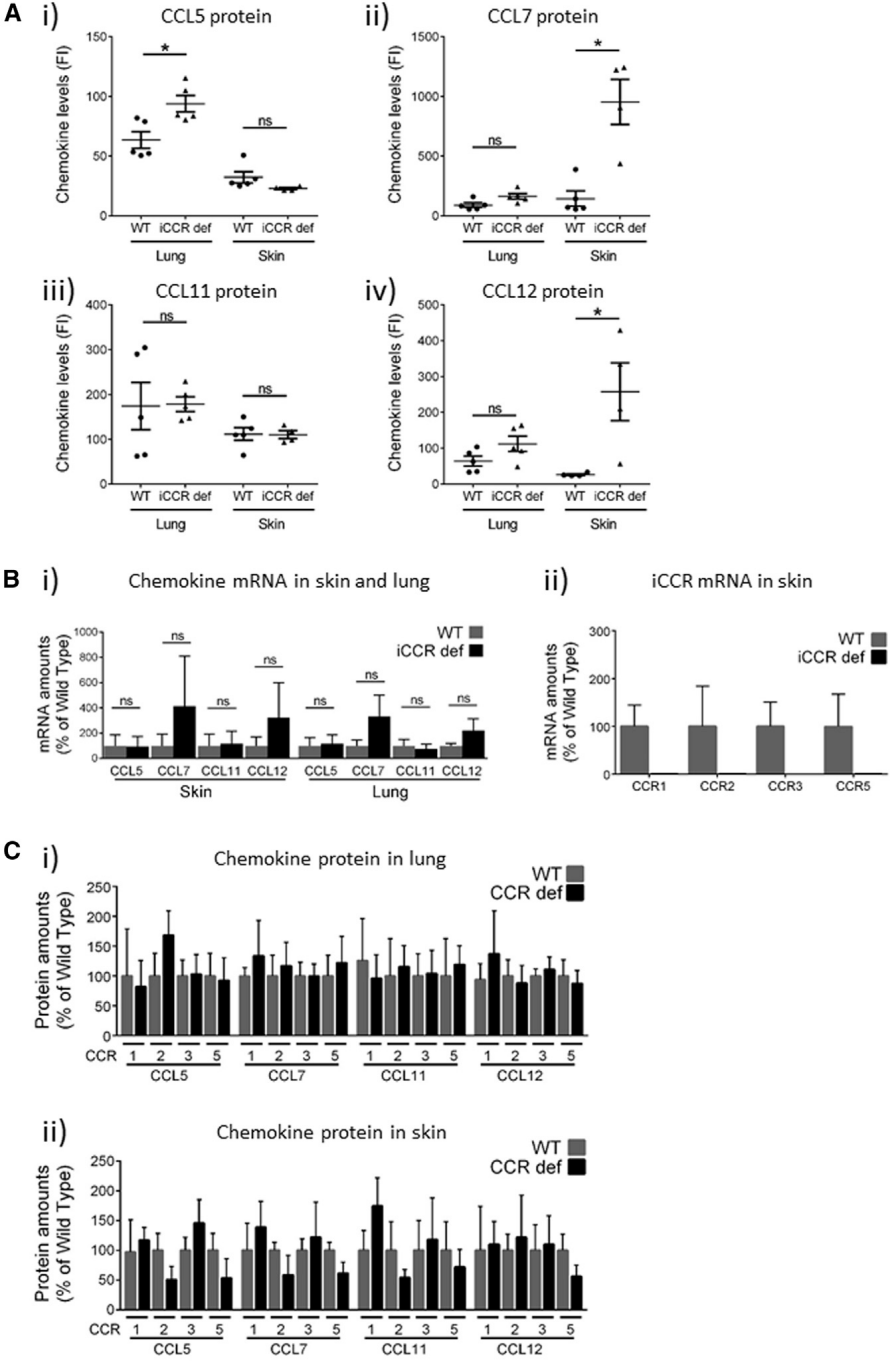
Chemokine Expression in Resting Skin and Lung (A) Concentrations of (i) Ccl5, (ii) Ccl7, (iii) Ccl11, and (iv) Ccl12 were measured in WT (n = 5) and iCCR-deficient (n = 5) lungs and skin via multiplex approaches. (B) (i) qPCR analysis of expression of *Ccl5, Ccl7*, *Ccl11*, and *Ccl12* in resting skin and lung. (ii) qPCR analysis of expression of *Ccr1*, *Ccr2*, *Ccr3*, and *Ccr5* in resting skin. (C) Analysis of the protein concentrations of the indicated chemokines in (i) the lungs and (ii) the skin of individual iCCR-deficient mice. Numbers represent mice deficiencies as follows: 1, CCR1 deficient; 2, CCR2, deficient; 3, CCR3 deficient; and 5, CCR5 deficient. No significant differences were noted between any of these data points. All numerical data in (A)–(C) are presented as mean + SEM. ^∗^p < 0.05; n.s., not significant. Data were analyzed with the Mann-Whitney U test. Each point represents an individual mouse.

The variability in receptor use apparent in our analysis of resting skin and lung could be explained by differential chemokine use in these tissues. Given that receptor function is associated with scavenging of cognate ligands (Cardona et al., 2008), we reasoned that elevated concentrations of i*Ccr* ligands in iCCR-deficient tissues would be indicative of cognate receptor involvement in leukocyte recruitment to these tissues. Using multiplexing analysis, we found that most chemokines were below limits of detection. However, as shown in Figure 4A, *Ccl5*, *Ccl7*, *Ccl11*, and *Ccl12* (*Ccr2* ligand) were detectable, and there was evidence of differential chemokine use between lung and skin. Specifically, and in agreement with a previous report (Galkina et al., 2005), elevated concentrations of *Ccl5* (Figure 4Ai) were detected in iCCR-deficient lung but not skin, suggesting that *Ccl5*-binding receptors are active in monocyte recruitment to resting WT lungs. In contrast, although *Ccl7* concentrations were not different between WT and iCCR-deficient lungs (Figure 4Aii), iCCR-deficient skin displayed significantly higher concentrations than WT skin. No differences were seen in *Ccl*11 concentrations (Figure 4Aiii), but again, although no differences in *Ccl12* concentrations were detected between WT and iCCR-deficient lungs (Figure 4Aiv), iCCR-deficient skin displayed elevated concentrations. Importantly, these differences in chemokine concentrations were not reflected in differences in transcript amounts (Figure 4Bi), confirming a role for receptor-scavenging rather than transcriptional induction in increased protein concentrations. In addition, all four i*Ccr*s were detectable in skin and lung (data shown for skin in Figure 4Bii), suggesting expression on tissue-resident leukocytes. Crucially, none of the chemokines tested displayed alterations at either the protein (Figures 4Ci and 4ii) or transcript (data not shown) levels in the lung and skin of individual iCCR-deficient mice. This indicates that raised concentrations in iCCR-deficient tissues are not a consequence of single-receptor deficiency but of combinatorial receptor use in steady-state monocyte recruitment to resting tissues.

Overall, these data indicate differential chemokine use in resting skin and lung. Of particular note is that *Ccl7* is known to bind to all of the i*Ccr*s (Bachelerie et al., 2014a), suggesting that its dominant use within the skin might account for the clear redundancy of receptor use in leukocyte recruitment to this tissue under resting conditions.

### Specificity in Receptor Use Is Evident for Recruitment of Cells to Sites of Acute Inflammation

**Figure 5:**
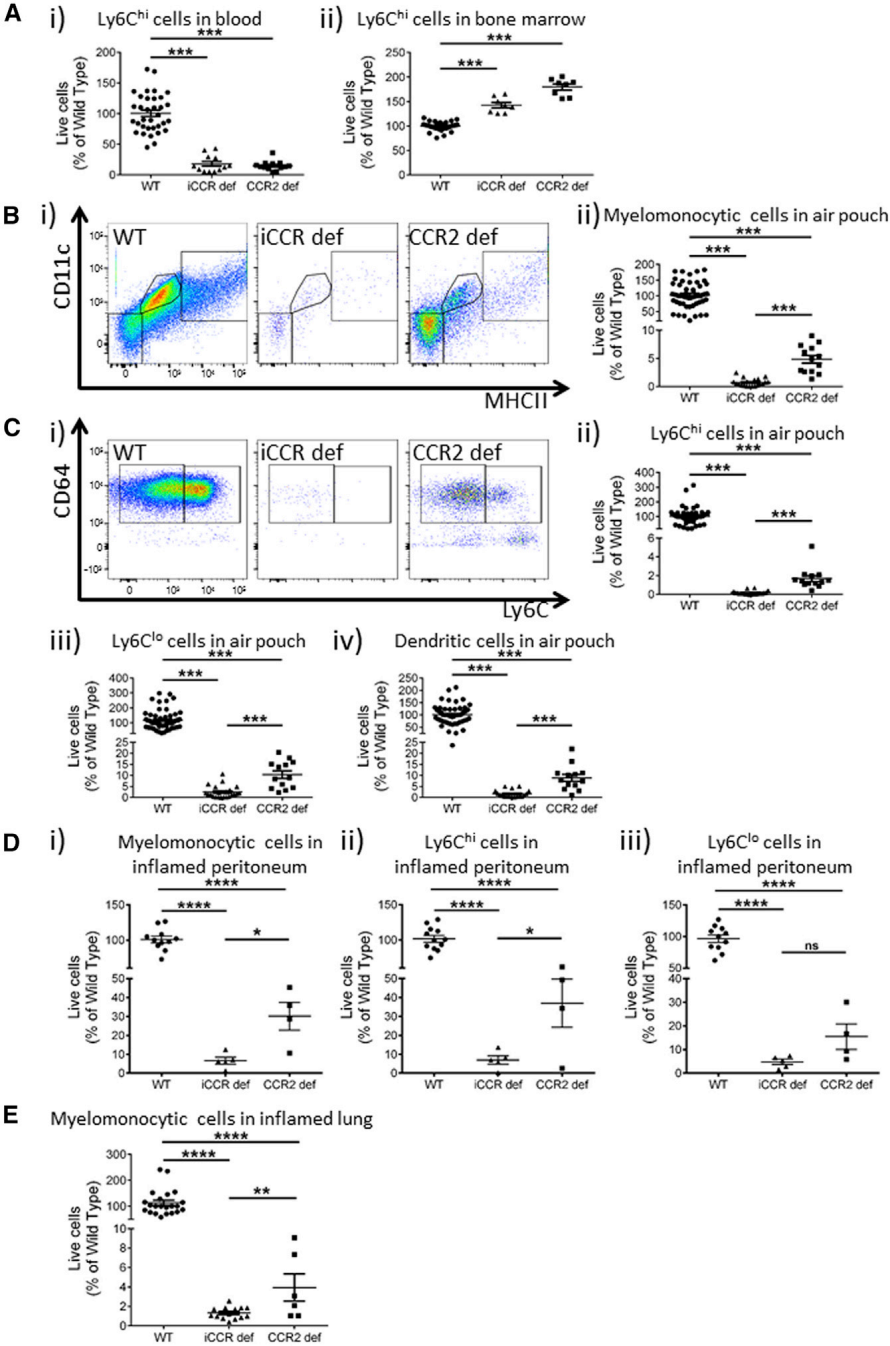
Myelomonocytic Cell Recruitment to Inflamed Air Pouches (A) Ly6C^hi^ cells in (i) blood (WT, n = 34; iCCR deficient, n = 12; CCR2 deficient, n = 13) and (ii) bone marrow (WT, n = 29; iCCR deficient, n = 8; *Ccr*2 deficient, n = 8). (B) (i) Flow-cytometric assessment of CD11c and MHCII expression in WT (n = 54), iCCR-deficient (n = 20), and CCR2-deficient (n = 13) cells recruited to the air pouch. (ii) Quantification of the percentage of the total monocytes and macrophages in the air pouch. (C) (i) Flow-cytometric assessment of CD64 and Ly6C expression on WT, iCCR-deficient, and CCR2-deficient myelomonocytic cells, as well as enumeration of (ii) Ly6C^hi^, (iii) Ly6C^lo^, and (iv) dendritic cells in the air pouch. Animal numbers as in (Bii) are shown. (D) Flow-cytometric evaluation of (i) total monocytes and macrophages, (ii) Ly6C^hi^ monocytes, and (iii) Ly6C^lo^ macrophages in the 24 h peritoneal exudate after intraperitoneal zymosan injection in WT, iCCR-deficient, and CCR2-deficient mice. (E) Flow-cytometric evaluation of total monocytes and macrophages in the lungs of WT, iCCR-deficient, and CCR2-deficient mice exposed to pulmonary influenza A virus infection. All numerical data in (A), (Bii), (Cii)–(Civ), (D), and (E) are presented as mean + SEM. ^∗^p < 0.05; ^∗∗^p < 0.01; ^∗∗∗^p < 0.001; ^∗∗∗∗^p < 0.0001. Data in (A)–(E) are compiled from at least three separate experiments and were log-transformed and analyzed by one-way ANOVA. Each data point represents a measurement from a single mouse. Please also see Figures S4 and S6 and Table S1.

To study leukocyte recruitment in inflammation, we used the air-pouch model (Colville-Nash and Lawrence, 2003, Edwards et al., 1981). This involved generating an air pouch in the mouse dorsum and injecting the inflammatory agent carrageenan into the air pouch. Subsequent sampling of the now inflamed air pouch (referred to simply as “air pouch” from now on) allowed precise analysis of recruited cells without the potentially confusing contribution from tissue-resident cells. 48 h after carrageenan introduction into the air pouch, the blood, bone marrow, and air-pouch contents were collected and analyzed for leukocyte content. Analysis of blood from iCCR-deficient and CCR2-deficient mice (Figure 5Ai) demonstrated equivalent Ly6C^hi^ monocytopenia (70%–80%) to that seen in resting mice. In contrast to the data from resting mice, Ly6C^hi^ monocytes accumulated in the bone marrow of iCCR-deficient and CCR2-deficient mice (Figure 5Aii) in this air-pouch model, indicating that they were expanded in number in response to the distant inflammation but were unable to efficiently leave the bone marrow because of the lack of *Ccr2* expression. Again, no differences in blood or bone marrow cellularity were noted in any of the other single-receptor-deficient mice (Table S1), including, in this inflamed context, CCR1-deficient mice. This demonstrates that *Ccr2* is also the sole i*Ccr* contributing to monocyte mobilization from the bone marrow in inflammation.

We conducted a broad-based analysis of myelomonocytic cell populations in the air-pouch by using CD11c and MHCII staining (as above), which revealed no differences in the recruited populations in CCR1- or CCR5-deficient mice. However, although we noted reductions in all three key populations in CCR2-deficient mice, we observed a complete block in recruitment of all three populations in the iCCR-deficient mice (Figure 5Bi). Quantification of total recruited monocyte and macrophage numbers confirmed these findings, demonstrating a 95% reduction in recruitment to the air pouch in CCR2-deficient mice but an absence of recruited monocytes and macrophages in iCCR-deficient air pouches (Figure 5Bii). Further, more detailed flow-cytometric analysis revealed reduced numbers of both Ly6C^hi^ and Ly6C^lo^ cells in CCR2-deficient air-pouches but a complete absence of these cells in iCCR-deficient air pouches (Figure 5Ci). This was confirmed by quantitative analyses, which revealed recruitment of approximately 2% Ly6C^hi^ and 10% Ly6C^lo^, as well as 10% dendritic cell, numbers in CCR2-deficient air pouches, compared with WT air pouches. All three cellular populations were essentially absent from iCCR-deficient air pouches (Figures 5Cii–5Civ). No differences in Ly6C^hi^, Ly6C^lo^, or dendritic cell numbers were noted for any of the other single-receptor-deficient mice (Table S1).

It is notable that CCR2-deficient mice have approximately 20%–30% of WT circulating Ly6C^hi^ cell numbers, but only 2% of cells displaying this phenotype are recruited to the air pouch. This raises two possibilities. First, small numbers of cells recruited via *Ccr2* express or induce chemokines, which amplify cellular recruitment via other receptors. Alternatively, these data could simply suggest that *Ccr2* is the dominant receptor in recruitment of myelomonocytic cells to acutely inflamed sites. To address these possibilities, we used multiplex approaches to analyze chemokine concentrations in air-pouch fluid. As shown in Figure S4C, we detected slightly higher concentrations of *Ccl3*, *Ccl5*, *Ccl12*, and *Cx3cl1* in air-pouch fluid in CCR2-deficient mice, suggesting reduced scavenging by their cognate receptors (*Ccr1*, *Ccr2*, *Ccr5*, and *Cx3cr1*) on monocytic cells and demonstrating that lack of recruited myelomonocytic cells does not lead to a reduction in chemokine concentrations within the air pouch. In fact, we detected no reduction in the concentrations of any detectable chemokines in the CCR2-deficient air pouch, indicating that “pioneering” cells that enter the air pouch and express chemokines are not required for subsequent leukocyte recruitment. Together, these data indicate that *Ccr2* is the dominant receptor not only for mobilization of Ly6C^hi^ cells from the bone marrow to the blood but also for recruitment to peripheral acutely inflamed sites.

Clearly, although useful for tissue sampling and analysis of leukocyte recruitment to inflamed sites in the absence of tissue-resident cells, the air-pouch model is artifactual given that the air pouch is not a physiological structure. Therefore, to complement these analyses, we also examined the response of WT, iCCR-deficient, and CCR2-deficient mice in models of intraperitoneal zymosan injection (Figure 5D) and pulmonary influenza A virus (IAV) infection (Figure 5E). The data revealed that, in both models, CCR2-deficient and iCCR-deficient mice again showed profound defects in myelomonocytic cell recruitment, and the block in recruitment was significantly greater in iCCR-deficient than in CCR2-deficient mice. This was shown for total monocytes and macrophages (Figure 5Di), Ly6C^hi^ cells (Figure 5Dii) and Ly6C^lo^ cells (Figure 5Diii) for the peritoneal model, and for total monocytes and macrophages (Figure 5E) for the IAV model. Together, these data confirm that the observations made from the air-pouch model also hold for more physiologically relevant models of inflammatory disease.

**Figure 6:**
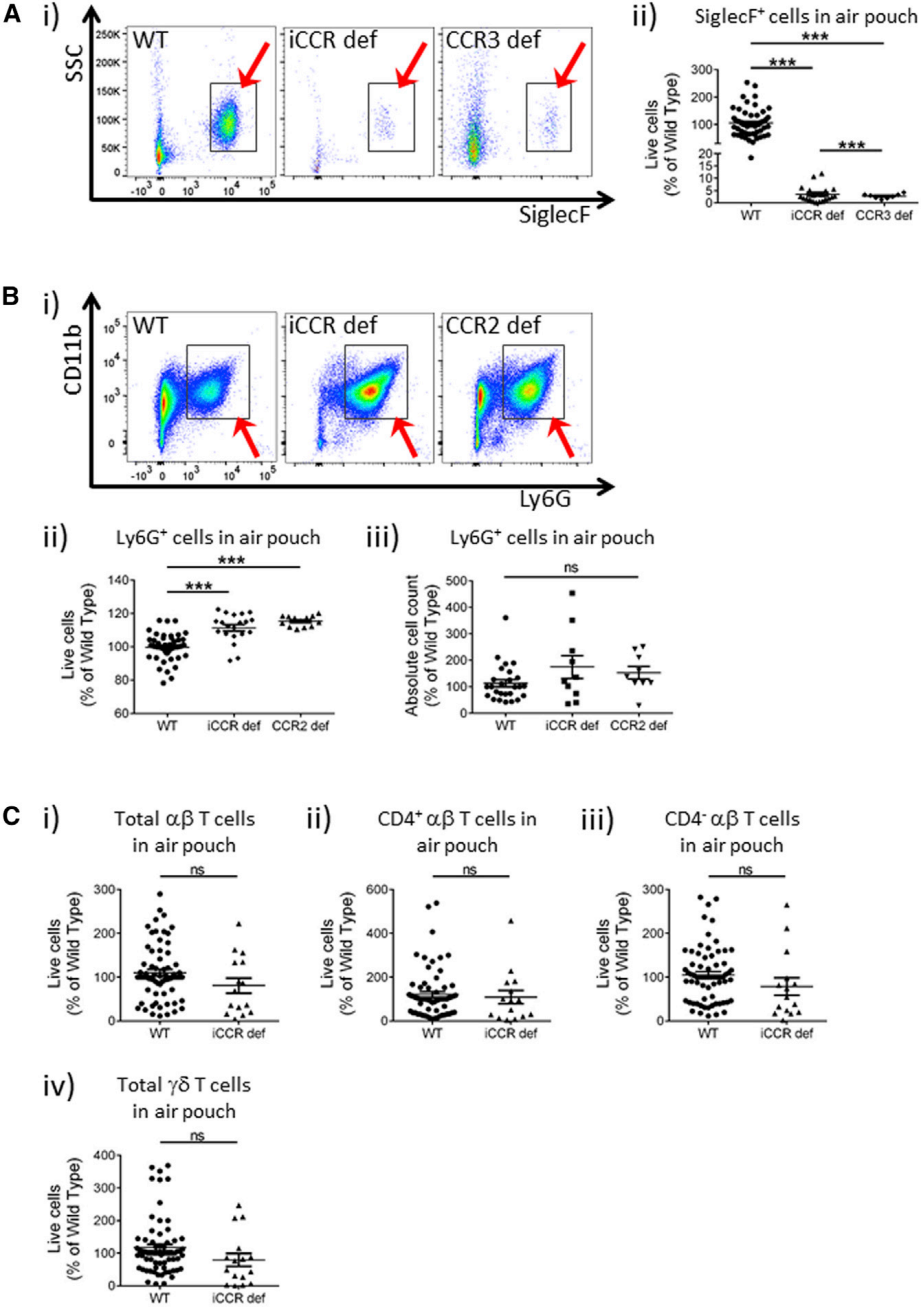
Eosinophil, Neutrophil, and T Cell Recruitment to the Air Pouch (A) (i) Flow-cytometric (eosinophils marked by the arrow) and (ii) quantitative assessments of eosinophils in WT (n = 63), iCCR-deficient (n = 20), and CCR3-deficient (n = 8) air pouches. (B) (i) Flow-cytometric (neutrophils marked by the arrow) and (ii) quantitative assessments of neutrophils in WT (n = 63), iCCR-deficient (n = 20), and CCR2-deficient (n = 13) air pouches and (iii) exemplar data showing absolute neutrophil numbers (expressed as a percentage of of WT numbers) in air-pouch tissue. (C) Enumeration of (i) total αβT cells, (ii) CD4^+^ αβT cells, (iii) CD4^−^ αβT cells, and (iv) γδT cells in WT (n = 69) and iCCR-deficient (n = 15) air pouches. All numerical data in (Aii), (Bii), (Biii), and (C) are presented as mean + SEM. ^∗∗∗^p < 0.0001; n.s., not significant. Data in (A)–(C) are compiled from at least three separate experiments and were log-transformed and analyzed by one-way ANOVA. Each data point represents a measurement from a single mouse. Please also see Figures S4 and S6 and Table S1.

Strong eosinophil recruitment was seen in WT, CCR1-deficient, CCR2-deficient, and CCR5-deficient air pouches (Table S1) but was essentially absent in iCCR-deficient and CCR3-deficient air pouches (Figures 6Ai and 6Aii). Notably, whereas no differences in neutrophil recruitment were detected in CCR1-, CCR3-, or CCR5-deficient mice, iCCR-deficient and CCR2-deficient air pouches apparently contained a proportionately higher neutrophil content than equivalent WT air pouches (Figures 6Bi and 6Bii). Importantly, this increase was not seen when the data were expressed as absolute numbers of neutrophils (Figure 6Biii), and this discrepancy was likely to be a consequence of a relative reduction in the size of other cellular populations in the iCCR-deficient and CCR2-deficient mice. Our data therefore provide no evidence of an active role for the i*Ccr*s in regulating neutrophil recruitment in acute inflammation. Finally, no significant differences in recruitment of any detectable lymphoid lineage cells were noted in iCCR-deficient mice or in any of the single-receptor-deficient mice (Figures 6Ci–6Civ). T helper 17 (Th17) cells were undetectable in the air-pouch model and in the lung tissues from mice undergoing IAV infection.

Overall, these data demonstrate that in this inflamed context, little if any redundancy of receptor use is apparent, and *Ccr2* is the dominant, non-redundant contributor to myelomonocytic cell recruitment to the air pouch. Our data further demonstrate combinatorial receptor involvement in overall myelomonocytic cell recruitment to inflamed sites and exclusivity of *Ccr3* involvement in eosinophil recruitment.

### The *Ccr2*-Independent Population Recruited to the Air Pouch Is Distinct from the Bulk Myelomonocytic Cell Population Recruited in WT Mice

**Figure 7:**
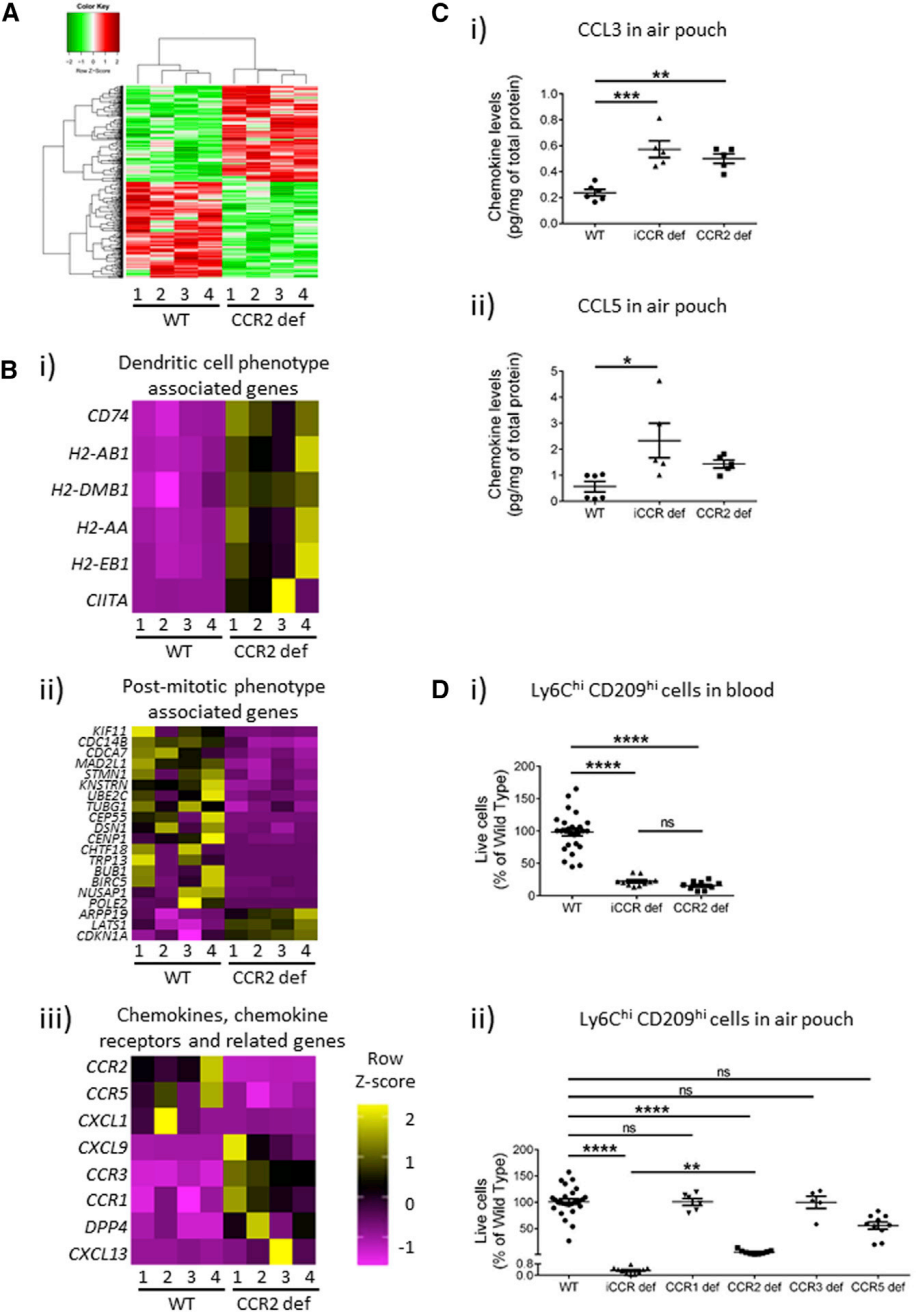
Transcriptomic Analysis of Myelomonocytic Cells in WT and CCR2-Deficient Air Pouches (A) Heatmap representation of the transcriptional differences between the bulk monocytic population recruited to WT air pouches and the small residual population recruited to CCR2-deficient air pouches. Green coloration represents downregulated genes, and red represents upregulated genes. (B) Heatmap comparison of (i) genes typical of a dendritic cell phenotype, (ii) genes indicative of a post-mitotic phenotype, and (iii) chemokine, chemokine-receptor, and related genes. In these heatmaps, yellow represents upregulated genes, and purple represents downregulated genes. (C) Chemokine concentrations in air-pouch fluid as measured by multiplex analysis (n = 5 per group). Data in (C) were analyzed with the Mann-Whitney U test. Note that the WT and *iCcr-*deficient data are the same as used in Figure S6 and are replicated here to highlight the point being made in the text. (D) (i) CD209a^hi^ cells in the blood of WT, iCCR-deficient, and CCR2-deficient mice. (ii) CD209a^hi^ cells in the air-pouch model in WT, iCCR-deficient, CCR1-deficient, CCR2-deficient, CCR3-deficient, and CCR5-deficient mice. All numerical data in (C) and (D) are presented as mean + SEM. ^∗^p < 0.05; ^∗∗^p < 0.01; ^∗∗∗^p < 0.001; ^∗∗∗∗^p < 0.0001; n.s., not significant. Please also see Figure S4 and Tables S1 and S2.

The Figure 5 data showing residual Ly6C^hi^ monocytic cell recruitment in CCR2-deficient air pouches suggest either that some classical Ly6C^hi^ myelomonocytic cells display redundancy of receptor use or that this residual population represents a *Ccr2*-independent, phenotypically discrete cell type. To address this, we isolated Ly6C^hi^ cells from the air pouch of WT and CCR2-deficient mice and compared their transcriptomic profiles, which indicated clearly (Figure 7A) that the residual recruited population in the CCR2-deficient air pouch was transcriptomically distinct from the bulk population recruited in WT mice. We observed 434 significantly differentially expressed genes, with 222 upregulated and 218 downregulated transcripts, between the two populations (Table S2). This residual population was characterized by high expression of *Cd209a* and increased expression of a range of transcripts involved in antigen presentation (Figure 7Bi). It, however, lacked the core dendritic cell gene set defined by the Immgen project (Miller et al., 2012) and therefore appeared not to be classical dendritic cells. Notably, this cellular population was also characterized by a reduction in genes involved in mitosis (Figure 7Bii) and therefore appeared to be post-mitotic. The generated transcriptomic data, particularly with respect to genes involved in antigen presentation, bore striking resemblance to those associated with a dendritic-cell-like sub-population of monocytes detected within the blood (cluster 3 genes in Menezes et al., 2016). Our data differ with respect to the presence of transcripts indicative of a post-mitotic state, suggesting that this monocyte-dendritic-cell population is rapidly terminally differentiated upon entering the air pouch. This population of cells is reported to be substantially dependent on *Ccr2* for mobilization from bone marrow to peripheral blood.

The transcriptomic data further indicated that the residual population in CCR2-deficient air pouches was characterized by elevated expression of *Ccr1* and *Ccr3* (Figure 7Biii). Notably, multiplex analysis of chemokine concentrations in air-pouch fluid revealed increased amounts of *Ccl3* and *Ccl5* (Figure 7C), further suggesting potential involvement of either *Ccr1* or *Ccr5* in recruitment of these cells. We examined this option by using single-receptor-deficient mice and pharmacological blockers of *Ccr1*. Our data clearly indicate, in agreement with the study mentioned above (Menezes et al., 2016), that this CD209a^+^ population is strictly dependent on *Ccr2* for effective egress from bone marrow (Figure 7Di). In terms of recruitment to the air pouch, again our data indicate a complete block to recruitment of these cells in iCCR-deficient mice but also a strong reduction in CCR2-deficient mice. This potentially indicates that the CD209a^+^ population is heterogeneous such that one subset is dependent on *Ccr2* for recruitment to acutely inflamed sites and one is independent of *Ccr2*. Notably, no other single-receptor deletion had any effect despite the apparent reduction in recruitment observed in CCR5-deficient mice, which did not reach statistical significance. Furthermore, a pharmacological blocker of *Ccr1* had no effect on recruitment of the CD209a^+^ population to air pouches in WT mice (data not shown). Overall, these data suggest a dominant role for *Ccr2* in bone marrow egress and, potentially, recruitment to the air pouch of this cellular population but also a residual role for the other i*Ccr*s, perhaps in a redundant fashion, in recruitment to the air pouch.

Overall, this transcriptomic analysis demonstrates that the cells recruited to the air pouch in CCR2-deficient mice are distinct from the bulk myelomonocytic cell population. Together, these data provide further evidence for the lack of redundancy in chemokine receptor involvement in inflammatory cell recruitment to air pouches and suggest combinatorial roles of the individual receptors in recruitment of the full complement of leukocyte subsets to acutely inflamed areas. Overall, our data indicate that i*Ccr* involvement in acute inflammation might be more precise and selective than previously thought.

## Discussion

There has been confusion and controversy regarding the roles for the i*Ccr*s in the regulation of myelomonocytic cell recruitment to inflamed sites (Mantovani, 1999, Schall and Proudfoot, 2011). With a view to defining the individual and combined roles for the i*Ccr*s in inflammatory cell recruitment, we generated mice in which the entire i*Ccr* locus had been deleted. Analysis of these mice, in comparison with single receptor-deficient mice, revealed evidence of apparent “redundancy” in recruitment of Ly6C^hi^ myelomonocytic cells to resting skin. None of the individual iCCR-deficient mice compared with WT mice displayed significant alterations in the numbers of these cells. However, the cells were severely depleted (70% depletion) in iCCR-deficient mouse skin. Quite how this apparent “redundancy” was manifest is not clear. It is possible that individual leukocyte subtypes simultaneously express multiple chemokine receptors, although, as noted below, *Ccr2* is clearly the dominant receptor on circulating monocytes. This might also be a stochastic process with sporadic expression of individual receptors at different time points supporting redundant patterning of the resting cell population over time. One other interesting feature of this resting tissue analysis is that, despite a profound monocytopenia in CCR2-deficient mice, there was no statistical difference in the numbers of Ly6C^hi^ cells in resting skin between these mice and WT mice. This suggests that the low number of recruited cells proliferate *in situ* to generate the full cellular complement (Hashimoto et al., 2013). It might also be, therefore, that this apparent redundancy is more related to recruitment of low numbers of cells expressing individual i*Ccr*s than to recruitment of cells expressing combinations of *Ccrs*, which then expand in number after recruitment to resting tissues. Although this is not redundancy per se, it would have the overall effect of similarly helping to establish the resting tissue monocyte population.

It is also notable that different tissues appear to utilize different i*Ccr*s and chemokines to establish resting leukocyte populations. In contrast to the skin, the spleen was fully dependent on *Ccr2* for establishment of its monocyte-derived cellular populations, whereas the lung showed a combined involvement of *Ccr1* and *Ccr2* in Ly6C^hi^ monocytic cell recruitment. The relatively high concentrations of *Ccl7* detected in resting iCCR-deficient skin suggested that it is a dominant recruiter of myelomonocytic cells in this context, and its ability to bind to *Ccr1*, *Ccr2*, *Ccr3*, and *Ccr5* could account for the observed redundancy of receptor use in this tissue. Given that increased *Ccl*7 concentrations are likely to be a consequence of a lack of scavenging through cognate receptor interactions in the iCCR-deficient tissues, the fact that concentrations of this chemokine were not altered in any of the single-receptor-deficient mice suggests that no individual receptor is dominant in its use in steady-state monocytic cell recruitment.

Despite the profound impact on myelomonocytic cell recruitment, our data provide no evidence to support a role for any of the i*Ccr*s in T cell recruitment to resting tissues. Therefore, in terms of populating resting tissues with leukocytes, receptors within the i*Ccr* locus appear to be exclusively involved in myelomonocytic cell and eosinophil recruitment, and there is clear evidence of a redundancy of receptor involvement in establishing skin-resident myelomonocytic cell populations.

Our data are also supported by an *in silico* analysis of i*Ccr* expression in individual leukocyte subsets. In our experience, many anti-murine chemokine receptor antibodies are of poor quality and do not give reliable and specific staining. However, we have mined the Immgen database (http://www.immgen.org), and it is clear that monocytic cells dominantly express *Ccr2* and have occasional low expression of *Ccr1* and *Ccr5.* In contrast, eosinophils express *Ccr3* at extremely high levels but display only weak transcription of *Ccr1*. No expression of i*Ccr*s, with the exception of *Ccr1*, was detectable on T cells (Figure S5B), although our data indicate that this appears to have no effect on their recruitment. Alveolar macrophages express only very low amounts of *Ccr1* and do not express *Ccr2*. This is in keeping with the fact that alveolar macrophage numbers are unaffected in CCR2-deficient mice.

In contrast to that in resting tissues, we found no evidence of redundancy in myelomonocytic cell recruitment to the air pouch. Our overall conclusion is that *Ccr2* is the dominant receptor for myelomonocytic cell recruitment to acutely inflamed sites and that, in its absence, this is profoundly impaired. However, we identified a subpopulation of recruited myelomonocytic cells, which were capable of migrating to the air pouch in a CCR2-independent manner and which appeared to be a dendritic-cell-like subpopulation. This population clearly had a dependency on *Ccr2* for egress from the bone marrow and potentially for recruitment into inflamed sites. In the absence of CCR2, at least a subset of these cells can also apparently utilize other iCCRs for recruitment to the air pouch. Our analysis of single-receptor-deficient mice and use of pharmacological blockers of CCR1 did not demonstrate clear involvement of any individual receptor in the CCR2-independent recruitment, and thus there is potential redundancy in iCCR involvement in recruitment of these cells to the air pouch. Overall, and in combination with the data from eosinophil analysis, there is no evidence of redundancy in receptor involvement in myelomonocytic cell recruitment to acutely inflamed sites. Indeed, our data argue for the overwhelming importance of *Ccr2* for both mobilization of monocytic cells from the bone marrow and recruitment to the acutely inflamed air pouch. Despite reports to the contrary in studies using alternative models (Contento et al., 2008, Lionakis et al., 2012, Reichel et al., 2006, Rot et al., 2013, Schaller et al., 2008), our data provide no evidence for a role for any of the iCCRs in the recruitment of neutrophils or T cell subsets to the air pouch, suggesting that their use might be context dependent.

In conclusion, this comprehensive analysis of iCCR involvement in leukocyte recruitment provides evidence of redundancy in resting cell recruitment to the skin but specificity of receptor use and primacy of *Ccr2* in recruitment of myelomonocytic cells to acutely inflamed sites. Our data further suggest that it could be worth examining *Ccr2* as a therapeutic target in appropriate acute rather than chronic inflammatory settings.

## STAR★Methods

### Key Resources Table

**Table:** 

REAGENT or RESOURCE	SOURCE	IDENTIFIER
**Antibodies**		

Anti-mouse CD11b (Clone M1/70)	eBioscience	Cat#47-0112-82; RRID: AB_1603193
Anti-mouse F4/80 (Clone BM8)	eBioscience	Cat#25-4801-82; RRID: AB_469653
Anti-mouse CD3e (Clone 145-2C11)	Biolegend	Cat#100308; RRID: AB_312673
Anti-mouse NK1.1 (Clone PK136)	Biolegend	Cat#108732; RRID: AB_2562218
Anti-mouse CD64 (Clone X54-5/7.1)	Biolegend	Cat#139309; RRID: AB_2562694
Anti-mouse MHCII (Clone M5/114.15.2)	Biolegend	Cat#107639; RRID: AB_2565894
Anti-mouse CD11C (Clone N418)	Biolegend	Cat#117310; RRID: AB_313779
Anti-mouse Ly6G (Clone 1A8)	Biolegend	Cat#127606; RRID: AB_1236494
Anti-mouse Ly6C (Clone HK1.4)	Biolegend	Cat#128024; RRID: AB_10643270
Anti-mouse CD45 (Clone 104)	Biolegend	Cat#109826; RRID: AB_893349
Anti-mouse CD103 (Clone M290)	BD Biosciences	Cat#562772; RRID: AB_2737784
Anti-mouse CD80 (Clone 16-10A1)	Biolegend	Cat#104729; RRID: AB_11126141
Anti-mouse SiglecH (Clone 551)	Biolegend	Cat#129606; RRID: AB_2189147
Anti-mouse CD209a (Clone MMD3)	Biolegend	Cat#833003; RRID: AB_2721636
Anti-mouse Siglec-F (Clone E50-2440)	BD Biosciences	Cat#552126; RRID: AB_394341
Anti-mouse CD19 (Clone eBio1D3 (1D3))	eBioscience	Cat#25-0193-82; RRID: AB_657663

**Bacterial and Virus Strains**		

IAV strain WSN	In house	N/A

**Chemicals, Peptides, and Recombinant Proteins**		

Collagenase D	Roche	Cat#11088858001
Dispase II	Sigma-Aldrich	Cat#D4693
DNase I	Roche	Cat#11284932001
Collagenase P	Roche	Cat#11213857001
Liberase	Roche	Cat#5401119001
Carageenan	Sigma-Aldrich	Cat#C1867
cOmplete™, Mini Protease Inhibitor Cocktail Tablets	Roche	Cat#04693124001
Zymosan A	Sigma-Aldrich	Cat#Z4250
Tissue Protein Extraction Reagent	ThermoFisher Scientific	Cat#78510

**Critical Commercial Assays**		

High capacity RNA-to-cDNA	Applied Biosystems	Cat#4387406
PerfeCTa® SYBR® Green FastMix	Quanta Biosciences	Cat#95073-012
Custom Mouse Magnetic Luminex Multiplex assay	R&D Systems	Cat#LXSAMSM- 16
Bio-Plex Pro mouse chemokine 33-plex	Biorad	Cat#12002231
Mouse RANTES INSTANT ELISA	ThermoFisher Scientific	Cat#BMS6009IN ST
Mouse MCP-3 INSTANT ELISA	ThermoFisher Scientific	Cat#BMS6006IN ST
Mouse MCP5 INSTANT ELISA	ThermoFisher Scientific	Cat#BMS6007IN ST
Mouse Eotaxin Platinum ELISA	ThermoFisher Scientific	Cat#BMS6008
BCA protein kit	Pierce	Cat#23227
Custom RT2 PCR Array	QIAGEN	Cat#330171
RT2 SYBR Green ROX qPCR Mastermix	QIAGEN	Cat#330520
RT2 First Strand Kit	QIAGEN	Cat#330404
RNeasy mini kit	QIAGEN	Cat#74104
RNeasy micro kit	QIAGEN	Cat#74004

**Experimental Models: Organisms/Strains**		

Mouse: CCR1def: B6.129S4-*Ccr1*t^m1Gao^	Gift from Dr. Takanori Kitamura	Taconic 4087; RRID: MGI:3614571
Mouse: CCR2def: B6.129S4-*Ccr2*^tm1Ifc^/J	The Jackson Laboratory	JAX 004999; RRID: IMSR_JAX:004999
Mouse: CCR3def: C.129S4-*Ccr3*^tm1Cge^/J	The Jackson Laboratory	JAX 005440; RRID: IMSR_JAX:005440
Mouse: CCR5def: B6.129P2-*Ccr5*^tm1Kuz^/J	Gift from Dr. Takanori Kitamura	JAX 005427; RRID: IMSR_JAX:005427
Mouse: iCCRdef: C57BL/6 (*iCcr*)^KO^	This paper	This paper

**Oligonucleotides**		

See Table S3 for primers used in this study	N/A	N/A

**Software and Algorithms**		

Cutadapt	Martin, 2011	N/A
Fastqc	N/A	http://www.bioinformatics.babraham.ac.uk/projects/fastqc/
Kallisto	Bray et al., 2016	N/A
DESeq2	Love et al., 2014	N/A
Heatmap.2 package	N/A	https://github.com/cran/gplots)
FloJo v10.4	Tree Star	https://www.flowjo.com/
Prism v7	GraphPad	https://www.graphpad.com/scientific-software/prism/

**Other**		

ACK lysis solution	ThermoFisher Scientific	Cat#A1049201
Fixable viability stain	eBioscience	Cat#65-0866-18
FcR blocking reagent	Milltenyi	Cat#130-092-575
Absolute count beads	ThermoFisher Scientific	Cat#C36950

### Contact for Reagent and Resource Sharing

Further information and requests for resources and reagents should be directed to and will be fulfilled by the Lead Contact, Gerard Graham (gerard.graham@glasgow.ac.uk).

### Method Details

#### Mouse generation and maintenance

Mice lacking the inflammatory CC chemokine receptor locus (i*Ccr*) encompassing *Ccr*1, *Ccr*2, *Ccr*3 and *Ccr*5 were generated in collaboration with Taconic Biosciences. In brief, LoxP sites were introduced by homologous recombination into the genomic DNA of ES cells, flanking the *iCcr* locus. The cluster was then deleted by Cre-mediated excision in C57BL/6 ES cells and deletion confirmed by PCR, using the primers detailed in Table S3. Targeted ES cells were then used to generate heterozygous mice, which were subsequently bred to homozygosity.

CCR1-deficient and CCR5-deficient mice were originally obtained from Taconic and Jackson labs respectively but were provided as a generous gift from Dr. Takanori Kitamura, University of Edinburgh. CCR2-deficient and CCR3-deficient mice were purchased from Jackson labs. CCR1-deficient, CCR2-deficient and CCR5-deficient were obtained and maintained on a C57BL/6 background, whereas CCR3-deficient mice were on a BALB/c background. All experiments were normalized to the appropriate WT controls. This involved comparison of all receptor-deficient mice on a C57BL/6 background with their precise congenic control and CCR3-deficient mice with BALB/c controls. All WT controls were derived from appropriate heterozygous crosses and maintained in the same animal house.

All mice were ‘rederived’ and housed in the animal facility of the Beatson Institute for Cancer Research and bred in a “specific pathogen free environment.” Routine genotyping of pups was undertaken by PCR analysis of ear samples (Transnetyx). All experiments were carried out under the auspices of a UK Home Office Project License and following ethical review by the University of Glasgow Ethics Review Committee.

#### Expression of the receptors in peripheral blood

Blood samples were taken from tail tips of resting WT or i*Ccr* heterozygous animals. After red blood cell lysis (ACK lysis solution, Thermo Fisher Scientific), whole RNA was extracted using the RNeasy mini kit with DNase treatment (QIAGEN). RNA was then reverse transcribed into cDNA using the High-Capacity RNA-to-cDNA Kit (Applied Biosystems) and cDNA was used in the analysis of *Ccr*1, 2, 3, 5 and *Cxcr*2 expression by QPCR (PerfeCTa® SYBR® Green FastMix, Quanta Biosciences). All QPCRs were performed in a Prism 7900HT Fast Real-Time PCR system (Applied Biosystems). i*Ccr* expression was calculated using standard curves specific for each gene and results were normalized to the expression of the housekeeping gene GAPDH. QPCR and standard primers used in these analyses are detailed in Table S3.

#### Resting tissue analysis

Mice “at rest” were culled and blood extracted from the vena cava, followed by perfusion using 20 mL of PBS (Sigma) containing 1mM EDTA (Sigma) before analysis of the cellular content of a number of tissues.

Dissected spleens were crushed onto 70 μm nylon mesh filters and washed with PBS (Sigma). Spleen and blood cell suspensions then underwent red blood cell lysis (ACK lysis solution, Thermo Fisher Scientific) before washing, ready for cellular content analysis. The serum from centrifuged blood was aspirated and taken for multiplex analysis as detailed below.

Shaved lower dorsal skin was dissected and chopped into fine pieces, followed by digestion in 4ml of digest cocktail (Hanks buffered saline solution (HBSS) containing collagenase D (1mg/mL Roche), dispase II (500 μg /mL, Roche) and DNase I (100 μg/mL, Roche)) for 1.5 hours at 37°C with shaking. Perfused lungs were dissected and chopped into fine pieces before digestion in 5ml of digest cocktail (RPMI containing DNase I (100 μg/mL, Roche), dispase II (800 μg /mL, Roche) and collagenase P (200 μg/mL, Roche)) at 37°C for 1.5 hours, with inversion after 45 min. Enzymes were neutralized by adding 20 μL of fetal bovine serum (FBS) to each tube before skin or lung cell suspensions were filtered through 40 or 70 μm nylon mesh filters, respectively, and washed for cellular content analysis.

#### Multiplex analysis of resting and air-pouch samples

Blood was extracted from the vena cava of resting or inflamed (air-pouch) mice as described above. Skin and lung were dissected from resting mice as described above. Air-pouch membrane and fluid contents were obtained as described below.

Blood samples were incubated for 30 min on ice and then centrifuged at 13000*g* for 20 min. Serum was collected and stored at −80°C until analysis. Skin, lung and air-pouch membrane were snap-frozen and ground in liquid nitrogen using a mortar and pestle. They were then processed as described below. Air-pouch fluid contents were centrifuged at 400 *g* for 5 min. Supernatant was collected and stored at −80°C until analysis. Skin, lung, air-pouch membrane and fluid cells were lysed in 0.5 mL of Tissue protein extraction reagent (T-PER, ThermoFisher Scientific) in the presence of protease inhibitor cocktail (Roche) by rotating at 4°C for 6 hours. Samples were then centrifuged at 10000 *g* for 5 min and supernatant collected and stored at −80°C until analysis.

Resting blood was analyzed using a customised Magnetic Luminex Multiplex assay (R&D Systems). Resting skin, lung and inflamed samples were analyzed using a Bio-Plex Pro mouse chemokine 33-plex (Biorad). All samples were read on a Luminex 200 machine (Biorad) in the Flow Cytometry core facility (III, Glasgow).

#### ELISA Analysis

Total protein was extracted from tissue samples as described under “Multiplex analysis of serum and air-pouch samples.” Total protein concentrations were determined by Pierce BCA Protein (ThermoFisher Scientific). Specific concentrations of Ccl5, Ccl7, Ccl11 and Ccl12 were measured by ELISA, using Mouse RANTES INSTANT ELISA (ThermoFisher Scientific), Mouse MCP-3 INSTANT ELISA (ThermoFisher Scientific), Mouse Eotaxin Platinum ELISA (ThermoFisher Scientific) and Mouse MCP5 INSTANT ELISA (ThermoFisher Scientific), respectively. BCA and ELISA assays were read out on a Sunrise™ microplate reader (TECAN). ELISA results were normalized to the concentration of total protein of each sample.

#### PCR array analysis

QPCR and standard primers used in these analyses are detailed in Table S3.

Chemokine expression in skin and lung were determined using Custom RT2 PCR Array plates (Qiagen). Plates were designed to cover the mRNA corresponding to the chemokines detected by Bio-Plex Pro mouse chemokine 33-plex plates (Biorad) used in the Luminex analysis. *Ccr*1, *Ccr*2, *Ccr*3, *Ccr*5 were additionally included in this assay. Reverse transcription, positive PCR and genomic DNA controls were used as quality controls and ActB and TBP as reference genes.

Samples of lung and skin were collected from WT or iCCR-deficient animals. Total RNA was isolated using the RNeasy mini kit (Qiagen), including an on column DNase digest, and served as template to generate cDNA with the RT2 First Strand Kit (Qiagen). qPCR Samples were set up with RT2 SYBR Green ROX Mastermix (Qiagen) and plates run on a Prism 7900HT Fast Real-Time PCR system (Applied Biosystems). Results were analyzed using the Qiagen Analysis Centre (Qiagen) and are shown as % of WT expression, normalized to ActB and TBP.

#### Air-pouch model of inflammation

The air-pouch model of leukocyte recruitment was utilized as described previously (Colville-Nash and Lawrence, 2003, Edwards et al., 1981). Sterile air (3ml) was injected subcutaneously into the mouse dorsum every 2 days on 3 occasions. 1 day after the final air injection, 1ml of autoclaved carrageenan (1% (w/v) in PBS, Sigma) was injected into the air-pouch. 48 hours later, mice were culled and blood extracted from the vena cava and prepared for analysis as described above. Air-pouches were flushed with 3ml of buffer (PBS containing 1mM EDTA and 1% (w/v) fetal bovine serum, Sigma) and lavage fluid was collected and incubated on ice until further analysis. The membrane surrounding the air-pouch was then dissected and digested for 1 hour at 37°C with shaking in 1ml of HBSS containing 0.44 Wünsch units of Liberase (Roche). Liberase was then deactivated by adding 20 μL of FBS and membrane cell suspensions were passed through 70 μm nylon mesh filters and washed. Blood, air-pouch lavage fluid and digested membrane samples were then analyzed for cellular content as described below.

#### Influenza A virus infection

Mice were briefly anesthetised using inhaled isoflurane and infected with 300 plaque forming units of IAV strain WSN in 20ul of PBS intranasally (i.n.). Mice were euthanised 8 days post-infection and lungs harvested for analysis. IAV was prepared and titered in MDCK cells.

#### Peritoneal inflammation model

The model of peritoneal inflammation was carried out as described previously (Dyer et al., 2017). Briefly, 1mg of zymosan in 200 μL PBS was injected into the peritoneum of the relevant mice, 24 hours later the peritoneum was flushed with 5mls of PBS containing 1% EDTA (weight:volume). Cells were washed in FACS buffer and the resulting cell suspensions stained for flow cytometry.

#### Flow cytometry staining and analysis

Cell suspensions were washed into PBS and stained for 20 min at 4°C using fixable viability stain (1:1000 in PBS, eBioscience). Samples were then washed in flow cytometry buffer (PBS containing 1mM EDTA and 1% FBS) and stained for 20 min at 4°C in 100 μL of antibody cocktail containing FcR blocking reagent (antibodies and FC blocking reagent indicated below, diluted 1:100). Cells were then washed again in flow cytometry buffer and fixed in 100 μL fixation buffer (BioLegend) for 20 min. After fixation, samples were analyzed on an LSRII or Fortessa flow cytometer (BD Biosciences) based in the Institute of Infection, Immunity and Inflammation flow cytometry core facility (University of Glasgow). Antibodies used: CD11b, F4/80, CD19 (eBioscience) CD3e, NK1.1, CD64, MHCII, CD11c, Ly6G, Ly6C, CD45, CD103, CD80, SiglecH, CD209a (BioLegend) Siglec-F and CD103 (BD Biosciences). FcR blocking reagent (MACS Miltenyi Biotec).

Flow cytometry data are expressed as % of WT. In brief, for each experiment, individual WT and receptor-deficient mouse leukocyte numbers (as % of Live) were normalized to the median of the WT congenic control values and expressed as a percentage (% of WT). We also compared data expressed in this way with data expressed as ‘absolute’ cell numbers and, with the exception of the data presented in Figure 6Bii and 6Biii, there were no differences relating to the manner of presentation.

#### RNA sequence analysis

Cell suspensions from the membrane of the inflamed air-pouch were prepared and stained with antibodies as described above. Live, CD45^+^CD11b^+^Ly6G^-^CD11c^-^CD3e^-^CD19^-^NK1.1^-^SiglecF^-^F4/80^+^CD64^+^Ly6C^+^ cells were sorted from WT or CCR2-deficient mice into RLT buffer using a FACSARIA II cell sorter (BD Biosciences). Total RNA was then isolated using an RNeasy micro kit with DNase treatment (QIAGEN) and stored at −80°C.

RNA analysis was undertaken by the Glasgow Polyomics Facility. Briefly, an Illumina TruSeq Stranded mRNA sample preparation kit was used to prepare sequencing libraries from total RNA, which were then sequenced (Illumina NextSeq 500).

Raw sequence reads were trimmed for contaminating sequence adapters and poor quality bases using the Cutadapt program (Martin, 2011). Bases that had an average Phred score of lower than 15 were trimmed and reads trimmed to less than 54 bases were excluded. Read quality was checked before and after trimming with the Fastqc program (http://www.bioinformatics.babraham.ac.uk/projects/fastqc/).

The reads were “pseudo aligned” to the transcriptome using the program Kallisto (Bray et al., 2016). The differential expression for the analysis groups was assessed using the Bioconductor package DESeq2 (Love et al., 2014). Heatmaps of the resulting data were generated in R using the heatmap.2 package (https://github.com/cran/gplots).

#### Statistical analysis

All statistical analysis was carried out using GraphPad Prism and all tests were 2-sided. For normally distributed data, 1-way ANOVA with Tukey’s post-test was used and for non-normally distributed data, Kruskal-Wallis analysis with Dunn’s post-test was carried out. Where marked differences in cell numbers were detected within experiments and where standard deviations followed accordingly, data were log-transformed prior to statistical analysis. In all analyses, p = 0.05 was considered the limit for statistical significance. In all figures, ^∗^ p < 0.05; ^∗∗^ p < 0.01; ^∗∗∗^ p < 0.001; ^∗∗∗∗^ p < 0.0001. Data are presented as mean ± SEM except for Table S1 which is mean ± SD.

### Data and Software Availability

The datasets generated during and/or analyzed during the current study are available from the corresponding author on reasonable request.

## Supplemental Information

Table S2

Document S2. Article plus Supplemental Information
