# Supplementary material for: Chemokine Receptor Redundancy and Specificity Are Context Dependent
Source: Immunity. 2019 Feb 19;50(2):378–389.e5. doi: 10.1016/j.immuni.2019.01.009 (PMC6382461; doi:10.1016/j.immuni.2019.01.009)
Supplement: Document S1. Figures S1–S7 and Table S1, S3 [file mmc1.pdf]

Immunity, Volume *50*

Supplemental Information

Chemokine Receptor Redundancy and Specificity Are Context Dependent

Douglas P. Dyer, Laura Medina-Ruiz, Robin Bartolini, Fabian Schuette, Catherine E. Hughes, Kenneth Pallas, Francesca Vidler, Megan K.L. Macleod, Christopher J. Kelly, Kit Ming Lee, Christopher A.H. Hansell, and Gerard J. Graham

Supplemental Information.

Dyer et al, Chemokine receptor redundancy and specificity are context dependent.

Table S2 (Related to Figure 7): List of genes differentially expressed between the bulk myelomonocytic cell population in WT air-pouches and the residual population in *Ccr*2-/- air-pouches.

THIS TABLE IS UPLOADED AS A SEPARATE FILE.

	WT	i*Ccr*-/-	*Ccr*1-/-	*Ccr*2-/-	*Ccr*3-/-	*Ccr*5-/-
**Blood**						
Ly6CHi	100±30.7	**41.1±17.8**	**62.9±16.0**	**34.8±17.9**	105±40.2	81±31.02
Ly6CLo	100±28.2	74.5±25.8	95.3±26.3	53.1±19.6	125±31.1	85±39.6
**Spleen**						
Ly6CHi	98.3±28.05	**27.7±21.01**	**67±16.85**	**24.8±12.38**	104.1±16.16	**38.0±19.26**
Ly6CLo	97.0±25.53	**49.9±33.63**	83.1±27.96	**52.47±30.9**	90.7±12.47	79.7±29.29
Eos	126.9±89.90	**828±274.8**	104±65.5	122.4±79.83	**719.2±343**	91.88±62.0
**Resting skin**						
Ly6CHi	97.7±32.08	**32.9±30.18**	84.1±47.06	85.97±70.83	62.78±26.07	77.14±46.23
Ly6CLo	105.4±47.82	**46.98±43.2**	196.5±107.6	**57.29±34.6**	131.8±43.33	138.9±58.23
DCs	102.1±34.30	**19.33±10.8**	113.1±38.19	**31.83±15.3**	77.81±35.02	100.5±27.85
Eos	109.7±54.66	**26.7±30.03**	167.7±86.04	195.9±109.6	**2.623±1.42**	115.7±72.07
**Resting Lung**						
Ly6CHi	98.49±29.97	**34.39±18.2**	61.73±26.88	**48.6±20.47**	87.66±32.06	89.24±38.23
Ly6CLo	98.42±28.56	85.9±31.93	98.36±28.60	71.32±48.36	93.29±31.08	98.72±24.42
DCs	100.5±22.02	139.1±71.04	102.7±23.87	186.0±99.77	77.88±25.47	113.3±35.54
Eos	112.5±58.60	**22.65±14.6**	**52.52±23.5**	**68.6±24.12**	113.3±65.58	71.04±30.84
Alveolar MΦs	105.3±27.48	106.4±76.19	108.8±21.46	150.2±114.6	88.49±35.62	103.1±28.64
**Airpouch**						
Ly6Chi	96.69±42.58	**0.17±0.19**	118.0±61.16	**1.68±1.14**	111.9±62.50	95.54±49.62
Ly6CLo	117.7±63.50	**2.46±2.76**	68.21±22.55	**10.39±6.20**	113.8±68.36	150.7±78.76
DCs	106±52.06	**1.623±1.67**	95.9±41.10	**8.86±5.68**	119.7±32.88	112.8±69.94
Eos	108.9±66.27	**3.48±3.21**	136.1±93.70	224.7±174.0	**2.72±0.33**	138.1±57.51
Neutrophils	99.76±7.08	111.3±8.54	95.72±8.37	115.4±3.14	96.11±9.53	102.4±7.84
**Airpouch Blood**						
Ly6CHi	100.8±31.29	**18.14±13.7**	120.4±34.89	**14.85±8.01**	119.6±39.59	116.8±62.96
**Airpouch Bone marrow**						
Ly6Chi	99.64±9.51	**142.6±15.7**	97.43±24.26	**180.1±17.4**	101.1±11.00	104.2±118.6

Table S1 (Related to all Figures): Leukocyte levels (as % of Live) for each WT and KO sample in each experiment were normalised to the median of the WT values in that experiment and expressed as a % (% of WT). Data for all experiments were compiled and the mean and SD for each leukocyte subset was calculated and is shown in this table. Since the original data are normalised to the medians of the WT values, the final means of the WT values do not necessarily equate to 100%.

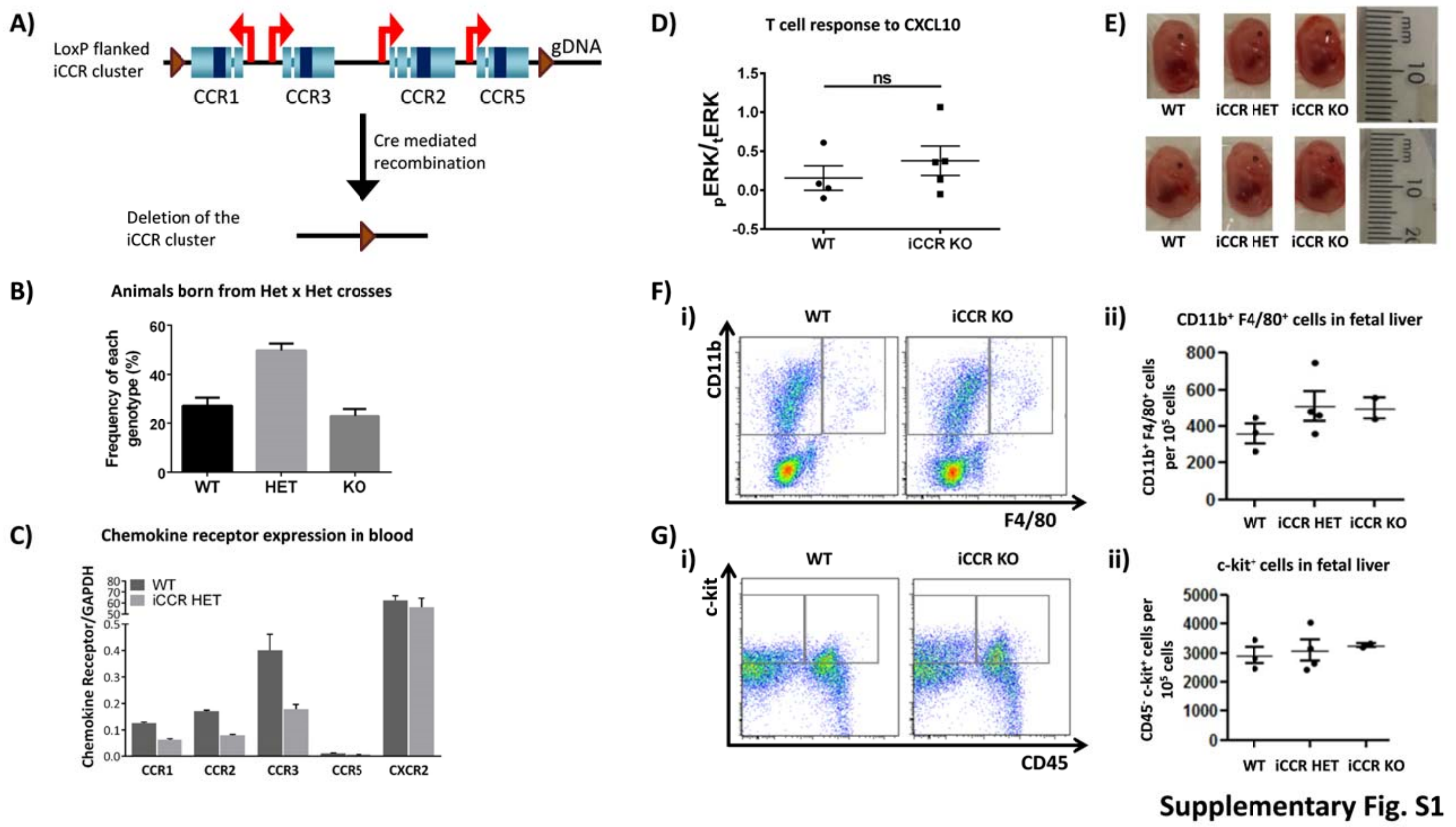

Figure S1 (Related to Figure 1).

A) The i*Ccr* locus spans approximately 170kb on mouse chromosome 9. It was deleted by insertion of 'loxP' sites at either end and Cre-mediated excision.

B) Frequency of WT, Het and KO offspring from Het-Het crosses.

C) Expression of the deleted receptors (*Ccr*1, *Ccr*2, *Ccr*3, *Ccr*5) in peripheral blood cells from heterozygous mice. Note that *Cxcr*2 acts as a control as this receptor is not deleted.

D) Densitometry of Western blot assessment of pERK levels, normalised to total Erk, in primary T cells from WT and iCcr-deficient mouse spleens in response to *Cxcl*10 (a Cxcr3 ligand).

E) Gross examination of WT, i*Ccr* heterozygous and i*Ccr*-deficient embryos at E14.5 (upper) and E15.5 (lower).

F) i) Flow cytometric and ii) quantitative analysis of $CD11b^{+}F480^{+}$ cellular populations in fetal liver from WT, i*Ccr*-heterozygous and i*CcrR*-deficient E15.5 embryos.

G) i) Flow cytometric and ii) quantitative analysis of $cKit^{+}CD45^{+}$ cellular populations in fetal liver from WT, i*Ccr*-heterozygous and i*Ccr*-deficient E15.5 embryos.

Each point in Figures Eii and Fii represents a single embryo (n=3-4). Data in E and F were analysed using the Kruskal-Wallis test with Dunn's post-test comparison.

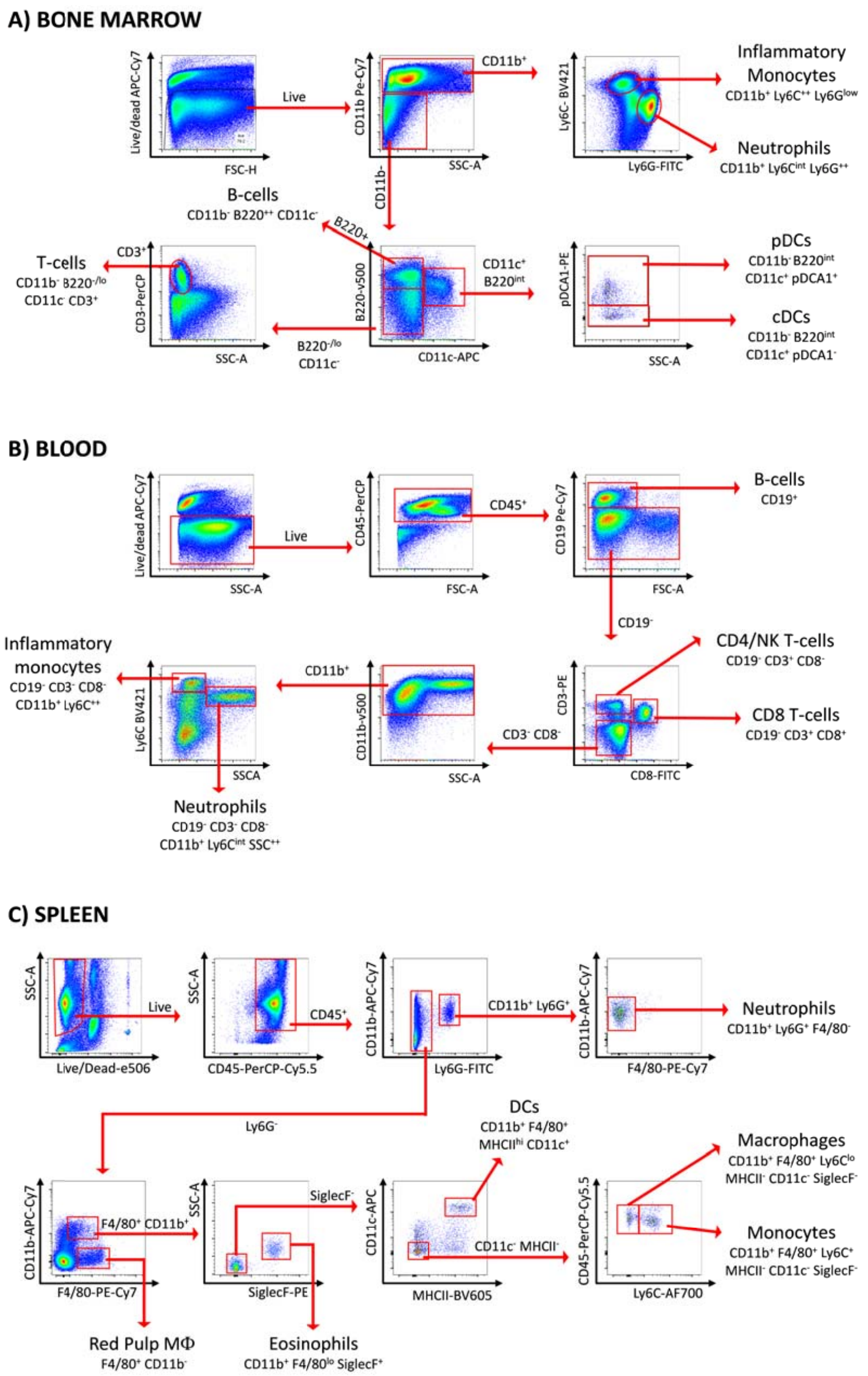

Figure S2 (Related to Figure 1). Gating strategies for Bone marrow, blood and spleen analyses.

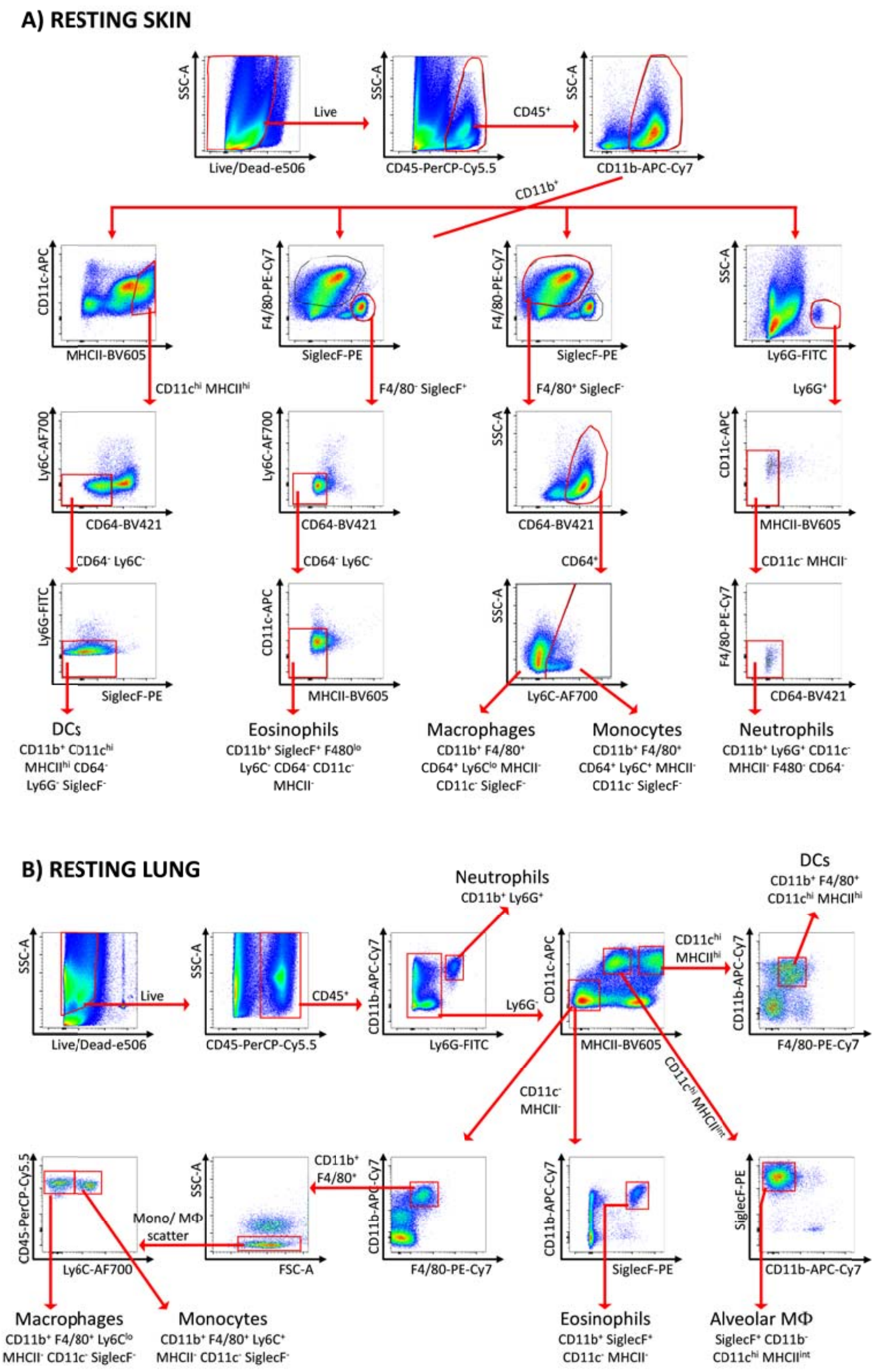

Figure S3 (Related to Figures 2 and 3). Gating strategies for skin and lung analyses.

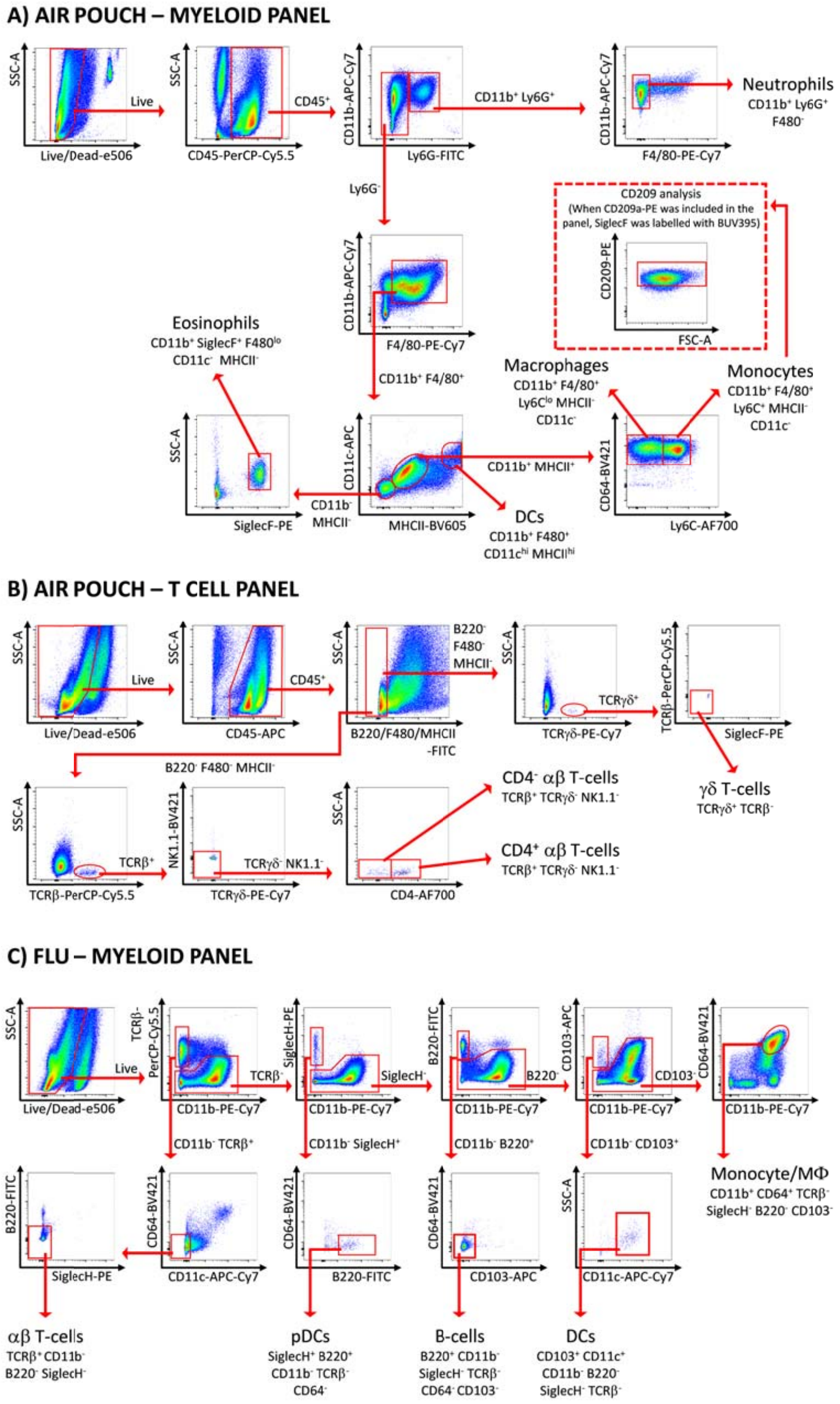

Figure S4 (Related to Figures 5, 6 and 7). Gating strategies for air pouch and flu analyses.

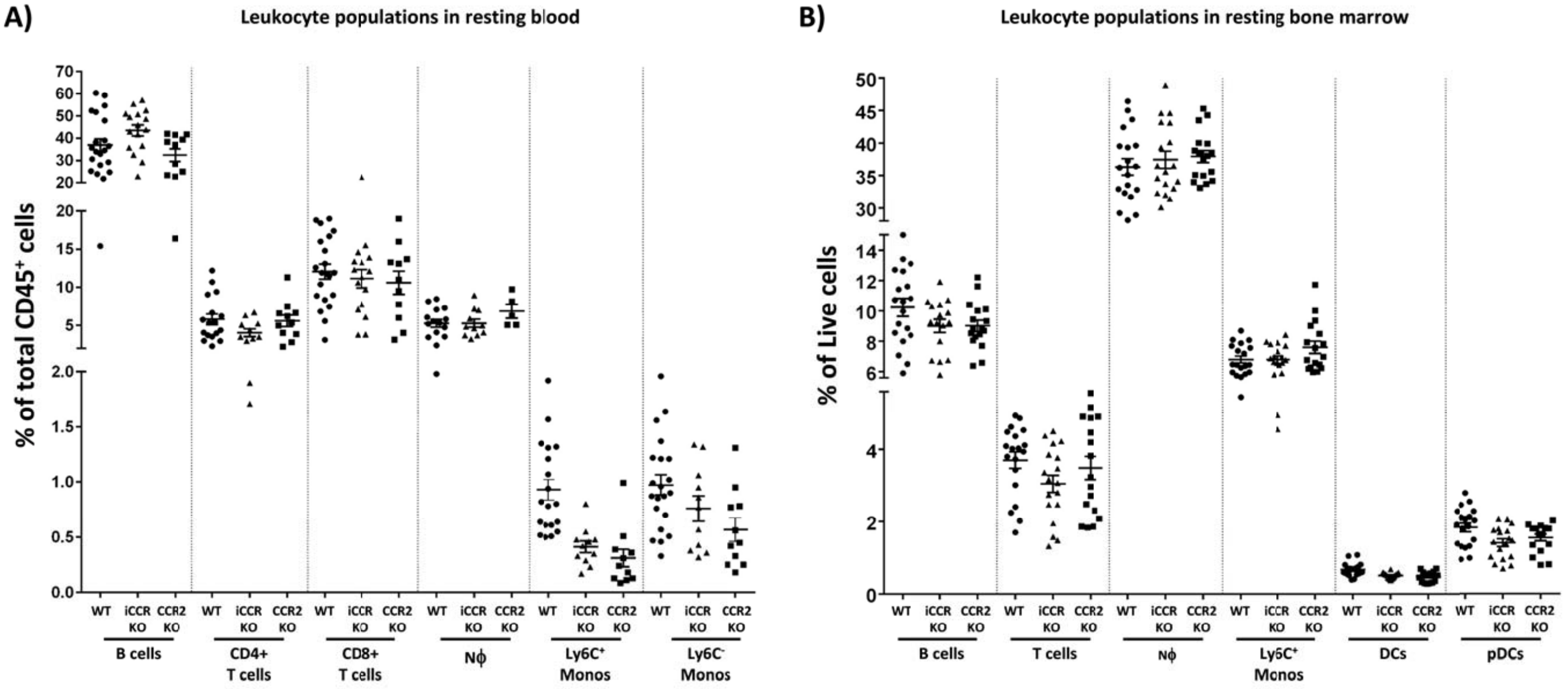

Figure S5 (Related to Figure 1): Comprehensive analysis of leukocyte populations in resting blood (A) and bone marrow (B) of resting WT, *Ccr*2-deficient and i*Ccr*-deficient mice. DCs-dendritic cells; pDCs-plasmamcytoid dendritic cells; NΦs-neutrophils. Data are expressed as percentage of CD45+ cells, for blood, and of live cells for bone marrow.

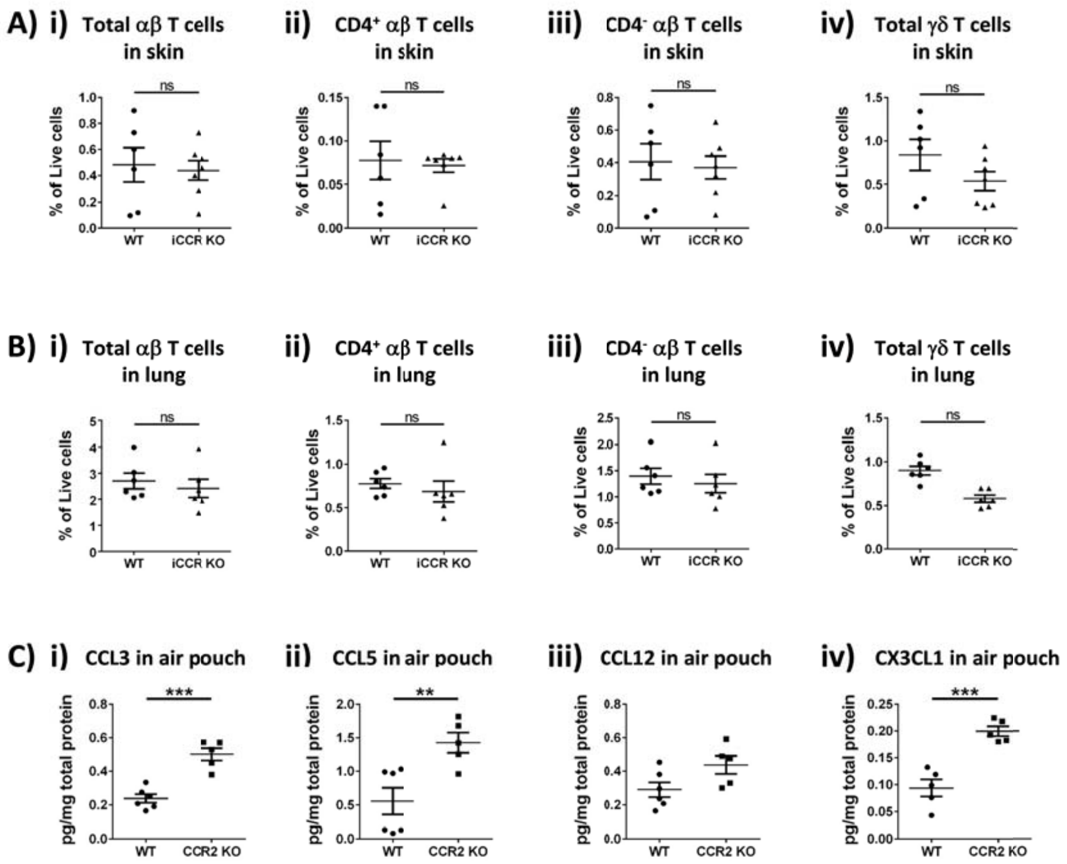

Figure S6 (Related to Figures 2, 3, 5 and 6): Data showing levels of i) total αβT cells; ii) CD4^{+} αβT cells; iii) CD4^{-} αβT cells and iv) γδT cells in resting skin (A) and lung (B). No significant differences were noted between WT and i*Ccr*-deficient mice for any of these lymphocyte subtypes. C) Data showing levels of chemokines in the air pouch of WT (n=5) and *Ccr*2-deficient (n=5) mice measured using multiplexing approaches.

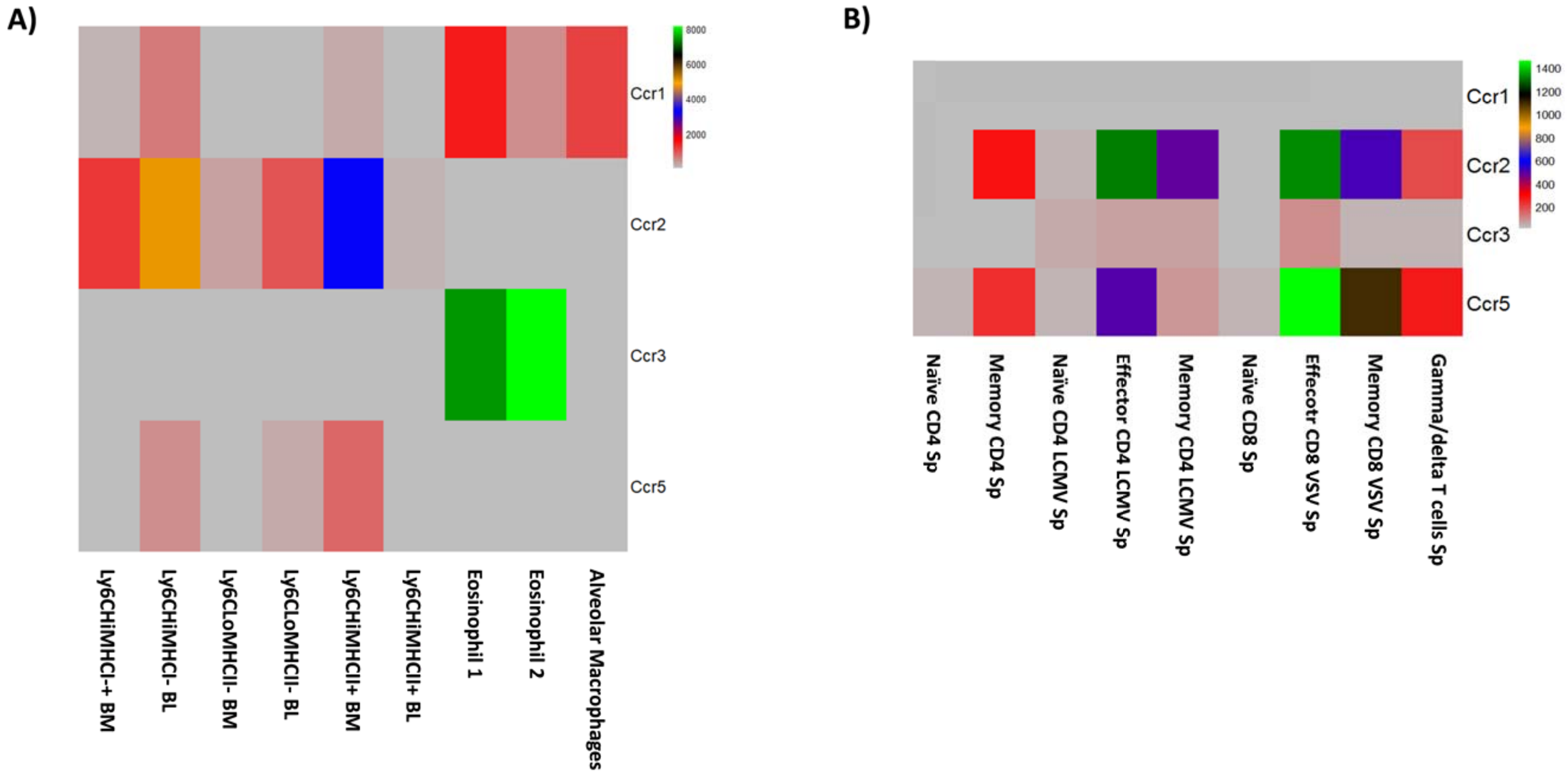

Figure S7 (Related to Figures 1, 2 and 3): In silico analysis of *iCcr* expression in A) monocytic, eosinophilic and alveolar macrophage populations and B) T cell subtypes, mined from the Immgen database (www.immgem.org).

Table S3. List of primers used in this study (related to STAR Methods).

iCCR-deficient mice Genotyping

WT1	CGTTGTTAGCCTCCTCATATCC
WT2	ACTGTTCCTTTAACTTCAGCTGG
WT3	GGGGCAATCAATTGAGGG
WT4	CAACCTCTGCTTGGTTCTGG
KO1	TTTCGACCGTCGTGGTTTG
KO2	TCATATCCAGTATGACAGACTTCAACC
KO3	GAGACTCTGGCTACTCATCC
KO4	CCTTCAGCAAGAGCTGGGGAC

Ccr **expression analysis**

Ccr1 QPCR1	GCCCTCATTTCCCCTACAA
*Ccr*1 QPCR2	CGGCTTTGACCTTCTTCTCA
*Ccr*1 Stan1	ACTTTTGGCATCATCACCAG
*Ccr*1 Stan2	CTCAGATTGTAGGGGGTCCA
*Ccr*2 QPCR1	TGTGGGACAGAGGAAGTGG
*Ccr*2 QPCR2	GGAGGCAGAAAATAGCAGCA
*Ccr*2 Stan1	AGGGGAGAGCAGAAGGCTAA
*Ccr*2 Stan2	CCCAGGAAGAGGTTGAGAGA
*Ccr*3 QPCR1	ACCTTCGGCTCTTTTTCCAC
*Ccr*3 QPCR2	TGTTCTTTCCATTTTCTCACCA
*Ccr*3 Stan1	GCCATCCGTCTTATTTTTGTTG
*Ccr*3 Stan2	ATTTCTTGCTCCCCAGTTGA
*Ccr*5 QPCR1	TTTGTTCCTGCCTTCAGACC
*Ccr*5 QPCR2	TTGGTGCTCTTTCCTCATCTC
*Ccr*5 Stan1	ACCCATTGAGGAAACAGCAA
*Ccr*5 Stan2	CTTCTGAGGGGCACAACAAC
*Cxcr*2 QPCR1	TGTCTGCTCCCTTCCATCTT

*Cxcr*2 QPCR2	CCATTTCCTCTCCTCCACCT
*Cxcr*2 Stan1	CGGGGTTCCTTCTTGTCTTT
*Cxcr*2 Stan2	TGCTATGTTCCTGTGTGAGG
GAPDH QPCR1	ATGTGTCCGTCGTGGATCTGAC
GAPDH QPCR2	GTTGCTGTTGAAGTCGCAGGAG
APDH Stan1	TGAACGGGAAGCTCACTGGC
GAPDH Stan2	TCCACCACCCTGTTGCTGTAG

qPCR Primers for *Ccl*5, *Ccl*7, *Ccl*11, *Ccl*12, *Tbp*

*Ccl*5 QPCR1	CTGCTGCTTTGCCTACCTCT
*Ccl*5 QPCR2	ACACACTTGGCGGTTCCTT
*Ccl*5 Stan1	CCCTCACCATCATCCTCACT
*Ccl*5 Stan2	TCAGAATCAAGAAACCCTCTATCC
*Ccl*7 QPCR1	TCAAGAGCTACAGAAGGATCACC
*Ccl*7 QPCR2	ATAGCCTCCTCGACCCACTT
*Ccl*7 Stan1	GCTGCTTTCAGCATCCAAGT
*Ccl*7 Stan2	AGAAAGAACAGCGGTGAGGA
*Ccl*11 QPCR1	GCACCCTGAAAGCCATAGTCT
*Ccl*11 QPCR2	TGGGGTCAGCACAGATCTCT
*Ccl*11 Stan1	TGCTGCTCACGGTCACTT
*Ccl*11 Stan2	CTTAGGCTCTGGGTTAGTGTCAA
*Ccl*12 QPCR1	AGCTACAGGAGAATCACAAGCA
*Ccl*12 QPCR2	TCCTTGGGGTCAGCACAG
*Ccl*12 Stan1	GACACTGGTTCCTGACTCCTCT
*Ccl*12 Stan2	AAGGTTCAAGGATGAAGGTTTG
TBP QPCR1	TGCTGTTGGTGATTGTTGGT
TBP QPCR2	AACTGGCTTGTGTGGGAAAG
TBP Stan1	GAGTTGCTTGCTCTGTGCTG
TBP Stan2	ATACTGGGAAGGCGGAATGT
